# Back to the future 2: the implications of germplasm structure on the balance between short- and long-term genetic gain in a changing target population of environments

**DOI:** 10.1093/g3journal/jkag044

**Published:** 2026-02-19

**Authors:** Frank Technow, Dean Podlich, Mark Cooper

**Affiliations:** Seed Product Development, Corteva Agriscience, Johnston, IA 50131, United States; Farming Solutions & Digital, Corteva Agriscience, Johnston, IA 50131, United States; Queensland Alliance for Agriculture and Food Innovation, The University of Queensland, St Lucia, QLD 4067, Australia

**Keywords:** biological complexity, adaptation, environmental change, breeding strategies, long-term genetic gain

## Abstract

Plant breeding operates within a complex genetic landscape determined by genes interacting within biological networks and with the environment. This environment is not constant but subject to short-term fluctuations and long-term shifts. This makes finding a balance between adapting germplasm for short- and long-term objectives challenging. We previously investigated the implications of genetic complexity on breeding program design. Here, we build on this work by adding an environmental dimension in the form of the E(NK) model to the simulation framework. We found that the addition of environmental interactivity and change creates greater uncertainty associated with pursuing any specific selection trajectory. This advantages preserving genetic variability and genetic landscape exploration over quickly exposing additive variation by constraining genetic space around a particular and temporary local optimum. Nonetheless, also in a dynamically changing environment, a distributed breeding program structure finds the best balance between short- and long-term objectives. In this structure, several breeding programs explore genetic space while maintaining constant germplasm exchange. This is in contrast to isolated programs or one large undifferentiated program, which exclusively emphasize short respectively long-term objectives. We furthermore highlight the difficulty of exchanging germplasm to restore genetic variability with nonstationary and germplasm context dependent genetic effects. In summary, also under environmental complexity and change, the structural features that characterized breeding operations hitherto and allowed them to navigating biological complexity apply. Namely, the necessity to constrain genetic space in order for heritable additive variation to emerge. We end by arguing that optimal breeding program design depends on the level of genetic and environmental complexity. This complexity should be appropriately reflected when modeling the long-term behavior of selection programs and the implications of specific interventions into these.

## Introduction

The productivity of crops, like all plant species, depends on the system of biotic and abiotic, natural and man-made, and environmental features they are embedded in (Comstock and Moll 1963; Hammer et al. 2006). (We use the term “environment” to refer to all extraneous factors influencing plant development, including agronomic practices.) The combinations of the various environmental factors are virtually endless. Individual environments are therefore often grouped into a smaller number of discrete environment types (ET), in which environmental conditions are relatively homogeneous (Gauch and Zobel 1997; Chenu 2015). The target population of environments (TPE) describes the frequencies of the different ET within a crop growing region (Comstock 1977; Cooper and Podlich 2002). Maximizing crop productivity across a TPE is one of the main concerns of farmers, agronomists, and plant breeders alike (Cooper et al. 2022). No single plant genotype can be optimally adapted to all ETs in a diverse TPE. This gives rise to genotype by environment interaction (GxE), ie the differential performance of genotypes across environments. GxE is one of the fundamental problems of plant breeding and considerable efforts have been and are undertaken to characterize, mitigate, and predict its effects (eg Comstock and Moll 1963; Cooper and DeLacy 1994; Piepho 1998; Elias et al. 2016; van Eeuwijk et al. 2016). The TPE is continuously changing as a result climate change (Chapman et al. 2012; Peng et al. 2020; Cooper et al. 2021), the evolution of weed, pest, and pathogens species (McCann 2020), or policy and economic shifts affecting agronomic practices (Kanter et al. 2020; Moore et al. 2023). Thus, not only is optimal adaptation across all ET within a TPE impossible, this adaptation itself is a moving target. Ensuring the adaptability of germplasm to a changing TPE is therefore an important concern of plant breeders and scientists alike (Kusmec et al. 2021; Xiong et al. 2022). Different approaches to this challenge have been proposed, such as utilization of gene bank resources (Mayer et al. 2020) and improved germplasm access and exchange (Galluzzi et al. 2020), accelerated variety development (Atlin et al. 2017), selection under conditions mimicking future environments (Cooper and Messina 2023), design of climate resilient ideotypes (Semenov and Stratonovitch 2013), and the use of gene editing technology (Karavolias et al. 2021). Like natural populations, plant breeding germplasm has an inherent ability to adapt to environmental change through recurrent selection (Becklin et al. 2016; Gao et al. 2023). A consequential decision breeders have to make is whether or not to stratify their germplasm into more or less independent subpopulations or programs and how much germplasm exchange to allow (Baker and Curnow 1969; Podlich and Cooper 1999; Cooper et al. 2014; Yabe et al. 2016; Ramasubramanian and Beavis 2021; Technow et al. 2021). (We use the term germplasm to refer to the standing genetic variation within the reference population of genotypes that is utilized in combination with the chosen breeding program structure to create and evaluate new genotypes according to breeding objectives.) In this study, we explore how breeding program structure affects the germplasm’s innate ability to create new genotypes with adaptation to environmental change and the balancing of adaptation to the current TPE (ie short-term genetic gain) with adaptation to potential future TPEs (ie long-term genetic gain). This is done in the context of hybrid varieties which emerged over a century ago (Shull 1908) and are the dominant variety type in most field and vegetable crops (Duvick 1999; Silva Dias 2010).

We have previously argued for the importance of considering the genetic complexity arising from highly interactive gene networks when studying the long-term behavior of selection programs in plant breeding (Cooper et al. 2005; Technow et al. 2021). One of the features of complex genetic systems is that gene effects are nonstationary and dependent on the genetic background and population context in which they are observed (Wade 2002; Cooper et al. 2005; Huang and Mackay 2016; Powell et al. 2021; Milocco and Salazar-Ciudad 2022). This also applies to derivatives such as the general combining ability (GCA) and specific combining ability, which are key measures in hybrid breeding (Sprague and Tatum 1942). The genetic context dependency is further compounded by the added complexity of environmental interactions. A trivial example is flowering time, where alleles conveying early flowering are beneficial in an early maturity environment but detrimental in a late maturity environment, and vice versa (Li et al. 2018). Therefore, we argue that it is necessary to consider the biological complexity resulting from interactions among genes and the environment to model and predict adaptation to long-term environmental changes (Podlich and Cooper 1999 ; Cooper and Podlich 2002). In this study, we build on the modeling framework previously developed by us (Technow et al. 2021) to investigate the implications of different breeding program structures on the balance between short- and long-term genetic gain in a changing TPE. We do this on the basis of the graph theoretical E(NK) model of trait genetic architecture (Cooper and Podlich 2002 ), which extents the *NK* model (Kauffman 1993) with an environmental dimension. Our primary objective is not to identify and recommend superior strategies for implementation in practice. Rather our aim is to propose a framework to investigate the short- and long-term behavior of selection programs under genetic complexity and environmental change.

## Materials and methods

### Model of genetic complexity and environmental change

The E(NK) model developed by Cooper and Podlich (2002) as an extension of Kauffman’s *NK* model (Kauffman 1993) will form the basis of the simulations. Here, *E* is representing the different ETs of the TPE, *N* (nodes in a graph) the number of genes for trait(s), as in the infinitesimal model, and *K* (edges in a graph) the average level of interactions among the *N* genes influencing the trait(s). Each E(NK) model comprises of a set of *NK* submodels, one for each ET. Varying the number of ET, the degree of similarity between the *NK* submodels, as well as the number of genes *N* involved in them and their degree of interaction *K*, creates a series of tunable models with differing environmental and genetic dimensionality and complexity. The use of the graph theoretical E(NK) framework for representing genetic architecture and its interaction with the environment was motivated by decades of research uncovering the biological networks underlying adaptation and output trait formation in globally important crops such as maize (eg Guo et al. 2014; Studer et al. 2017; Simmons et al. 2021; Tomura et al. 2025), rice (eg Wilkins et al. 2016), and wheat (eg Harrington et al. 2020).

#### E(NK) model implementation

We will first describe the *NK* submodels and then how these were integrated into the E(NK) ensemble. The *NK* submodels were implemented as in Technow et al. (2021), following the generalized approach described by Altenberg (1994), with the necessary adaptations for diploid genomes. Briefly, the complex trait is described as sum of a set of *fitness components*, normalized by dividing by *N* out of convention (Csaszar 2018). The values of the fitness components are distributed uniformly between 0 and 1 and are calculated as *random functions* of the genotypes at *K* interacting genes drawn at random from all *N* genes. The random functions were derived from the *ran4* pseudorandom number generator (Press et al. 1992). As in Technow et al. (2021), the number of fitness components and the number of genes were both set to N=500. A number that reflects the very large number of genes and metabolites that are involved in development, plant architecture and biomass metabolism of complex organisms such as maize (eg Saha et al. 2011; Powell et al. 2022) while still remaining computationally tractable. Genes were biallelic and the simulated organism diploid. The range of the complexity parameter *K* considered was from 1 to 15 in steps of 1. For values of *K* > 1 we allowed for some variation in the number of interacting genes by sampling the gene number of each component from a Poisson distribution with rate parameter equal to *K* and then truncating the sampled values to fall between 1 and 15. At K=1, all fitness components were controlled strictly by a single gene. For *K* > 1, genes were assigned at random to the fitness components, and individual genes consequently were typically involved in multiple fitness components, ie they acted pleiotropically. For K=1, each of the 500 genes was assigned to exactly one fitness component at random and the values of heterozygous genotypes set to be midway between the alternate homozygous genotypes. Thus, at K=1 genes acted strictly additively and without any intra- or intergene interactions. Because the expected value of the maximum of two samples from a Uniform distribution between 0 and 1 is 2/3, the expected maximum attainable fitness at K=1 is also 2/3. The complexity of the resulting genetic space was quantified in our previous work (Technow et al. 2021).

**Fig. 1.**
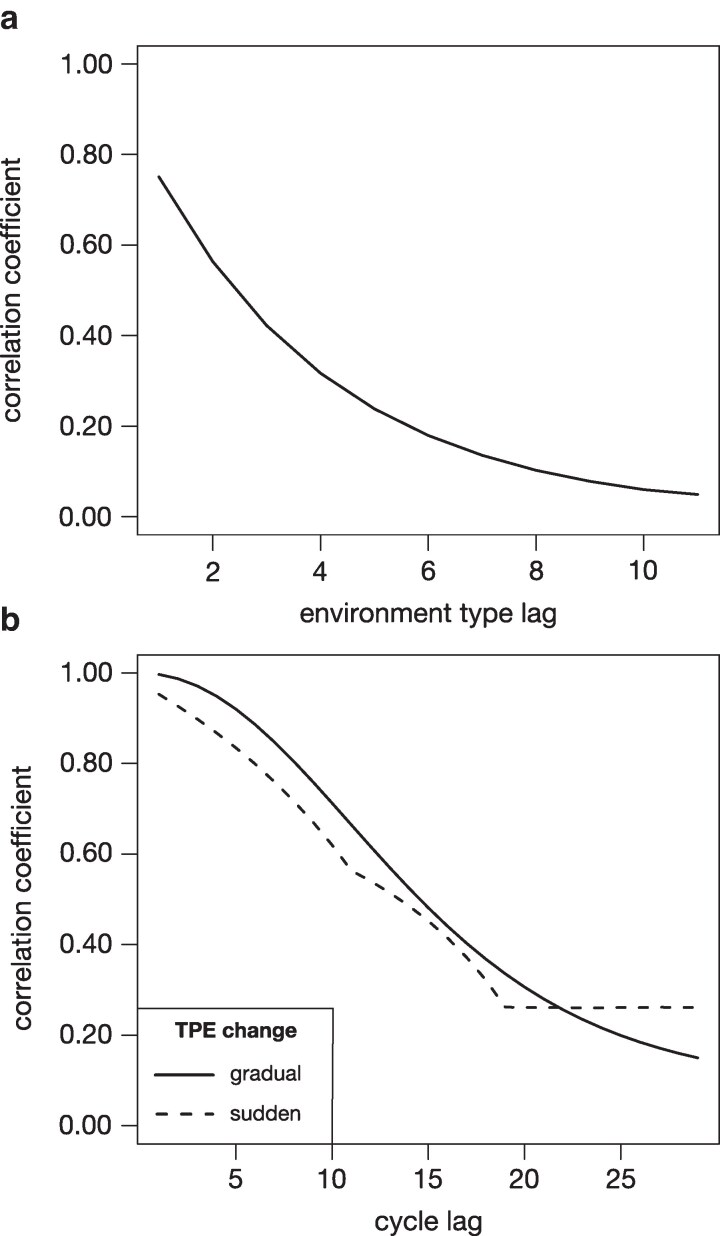
a) Average correlations between the ET specific performance as a function of ET lag. b) Average correlations between across TPE performance lagged across cycles.

The complete *E(NK)* model was comprised of 12 *NK* submodels representing 12 discrete ET. This number was again a compromise between the large number of possible combinations of environmental factors (including crop management and pathogen and pest pressure) that can give rise to differential adaptation requirements, the typical number of ETs defined and targeted by a breeding program (eg Löffler et al. 2005), and computational tractability. The ET were assigned numbers from 1 to 12 on an ordinal scale. We will henceforth refer to the number distance between these numbers as “lag” (eg ET3 and ET6 have a lag of 3). The *NK* submodels underlying each ET were created in such a way that the genetic correlation between the genotypic values in different ET, a common measure of the magnitude of GxE (Falconer 1952 ; Cooper and DeLacy 1994), decreased with increasing ET lag. To achieve this, 75% of the fitness components of each *NK* submodel were retained unchanged from the submodel of the previous ET and 25% were assembled anew as described above. Being the first in the series, the submodel for ET1 was assembled completely from scratch. Thus, ETs with a lag of 1 will have 75% fitness components in common, ETs with a lag of 2 about 50%, and so on. To directly quantify the resulting GxE correlation, 250 genotypes were generated at random and their fitness values calculated for each ET. The resulting correlation matrix was then summarized by averaging pair-wise ET correlations with the same lag. This process was repeated 300 times, each time using a new E(NK) model and 250 newly created genotypes. This was done at the intermediate value of K=6, though preliminary analysis showed that results were consistent across all values of *K* (results not shown). The average GxE correlation by ET lag is shown in Fig. 1a. The genetic correlation between directly neighboring ET (lag=1) was about 0.75, it dropped below 0.25 at around a lag of 5 and ET with a lag above 7 were virtually uncorrelated.

#### TPE change scenarios

**Fig. 2.**
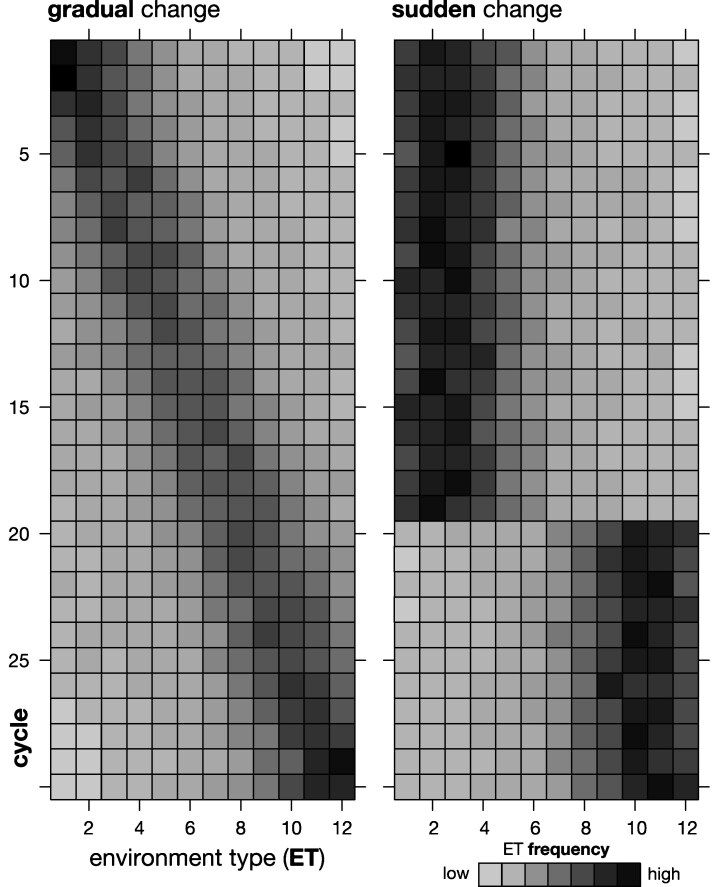
Visualization of change in ET frequency distribution for representative examples of the gradual (left) and sudden (right) TPE change scenarios.

A TPE can be described as a frequency distribution of ET (Cooper and Podlich 2002). Environmental change can thus be modeled as a change in those frequencies. Because the ET were modeled to be on an ordinal scale, this can conveniently be done by shifting the center (ie the area of highest probability weight) of the frequency distribution. We considered two environmental change scenarios taking place over 30 breeding cycles (i) *gradual* change and (ii) *sudden* change. Under gradual change, the center of the ET distribution shifted gradually from ET1 in the first breeding cycle to ET12 in the last cycle (Fig. 2). The most prominent example of this scenario are the incremental environmental changes brought about by climate change, such as the slow but steady rise in temperature and ${\text{CO}}_{2}$ levels (Cooper et al. 2021). In the sudden change scenario, the weight of the frequency distribution remained centered over ET 1 to 6 for 20 breeding cycles and then shifted abruptly to being centered over ETs 7 to 12 for the remaining ones. This scenario represents the emergence of new pathogens and pests (McCann 2020), irrigation resources like the threatened Ogallala Aquifer in the western US corn belt reaching a critical depletion level (Basso et al. 2013) or enactment of new agricultural policies, like those regulating the application of nitrogen fertilizer (Kanter et al. 2020).

The actual ET frequencies were sampled from Dirichlet distributions with concentration parameters chosen such that the center of the ET frequency distribution followed the general TPE change pattern outlined above. Sampling rather than setting these frequencies directly mirrors random year over year variability, in eg weather patterns. Because the support of the Dirichlet distribution is strictly positive, all ET had nonzero frequencies. The majority of the frequency distribution, however, was concentrated over a few ET around the current center of the distribution. This can be quantified with the “effective number” of ET, calculated as $1/b^{\prime }b$, where b is the vector of ET frequencies. This corresponds to the “Inverse Simpson Index” concentration measure used in eg ecology. The average values were 5.9 and 5.4. for the gradual and sudden TPE change scenarios, respectively. Thus, less than half of all 12 ET had an effective presence in the TPE at any given point in time.

True performance values of individuals in a given TPE were calculated as the average fitness values across the separate ET, weighted by their frequencies in the current TPE. The GxE correlation between TPE performance values across different cycles was calculated similarly as the GxE correlation between ET, ie from the true performance values of 250 randomly generated genotypes across all 30 cycles at a complexity level of K=6. This was also repeated 300 times with newly generated genotypes and E(NK) models. Results are shown in Fig. 1b. Consistent with expectations, the GxE correlation gradually decreased with increasing cycle lag in the gradual TPE change scenario. The lag curve for the sudden change scenario is included for completeness, but it is less meaningful as a descriptor of the GxE correlation between cycles in this scenario. Here, the only directional change in the TPE occurred at cycle 20. Consequently, the GxE correlation between the TPEs in cycles 1 to 20 and 21 to 30 was close to one and would have been equal to one if not for the small random frequency differences imposed. The GxE correlation between cycles with different TPEs (eg between cycle 8 and 23) was 0.266 and similar in magnitude to the GxE correlations between the largest cycle lags in the gradual scenario.

**Fig. 3.**
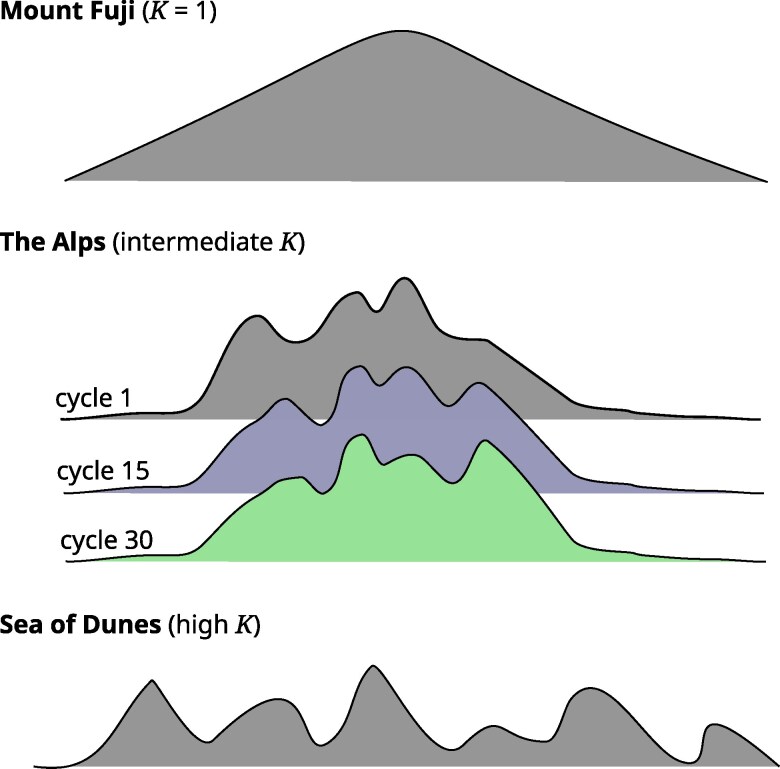
Schematic visualization of genetic landscapes corresponding to different values of complexity parameter *K*. The different colors in the “The Alps” schematic represent the changing genetic landscape resulting from environmental change over cycles. Figure modified with permission from Fig. 2 of Technow et al. (2021).

Wright (1932) introduced the metaphor of a *genetic landscape* as a conceptualization of high-dimensional genetic complexity. Naturally, it has also been found useful for developing an intuition for the ruggedness of different *NK* models (eg Kauffman and Weinberger 1989). While it should be emphasized that as a metaphor it should not be taken literally, we too find it helpful in making accessible the rather abstract concepts surrounding the discussion of the properties of E(NK) models. Using the terminology of Technow et al. (2021), the landscape at K=1, where genes act strictly additive without any inter- or intragene interactions, can be visualized as *Mount Fuji*, ie an isolated peak with monotonous incline to the top (Fig. 3). At intermediate levels of *K*, the genetic landscape contains a cluster of peaks, like a mountainous region in an otherwise flat landscape, similar to the *European Alps*. Finally, at high values of *K*, the landscape can be thought of as a *Sea of Dunes*, ie an endless number of evenly distributed peaks of similar shape and size. The different E(NK) submodels representing the ET would then correspond to a series of different landscapes with more or less similar features. And TPE change in particular could be thought of as landscape change, with old peaks flattening and new ones appearing as a result of geological forces.

#### Genome definition

The simulated genome consisted of 10 diploid chromosome, each of 1 Morgan in length and with the N=500 genes distributed evenly across them. Meiosis and recombination was simulated according to the properties of the Haldane mapping function with the R package *hypred* (Technow 2013), which is available from the supplement of Technow and Gerke (2017).

### Simulation of hybrid breeding process

**Fig. 4.**
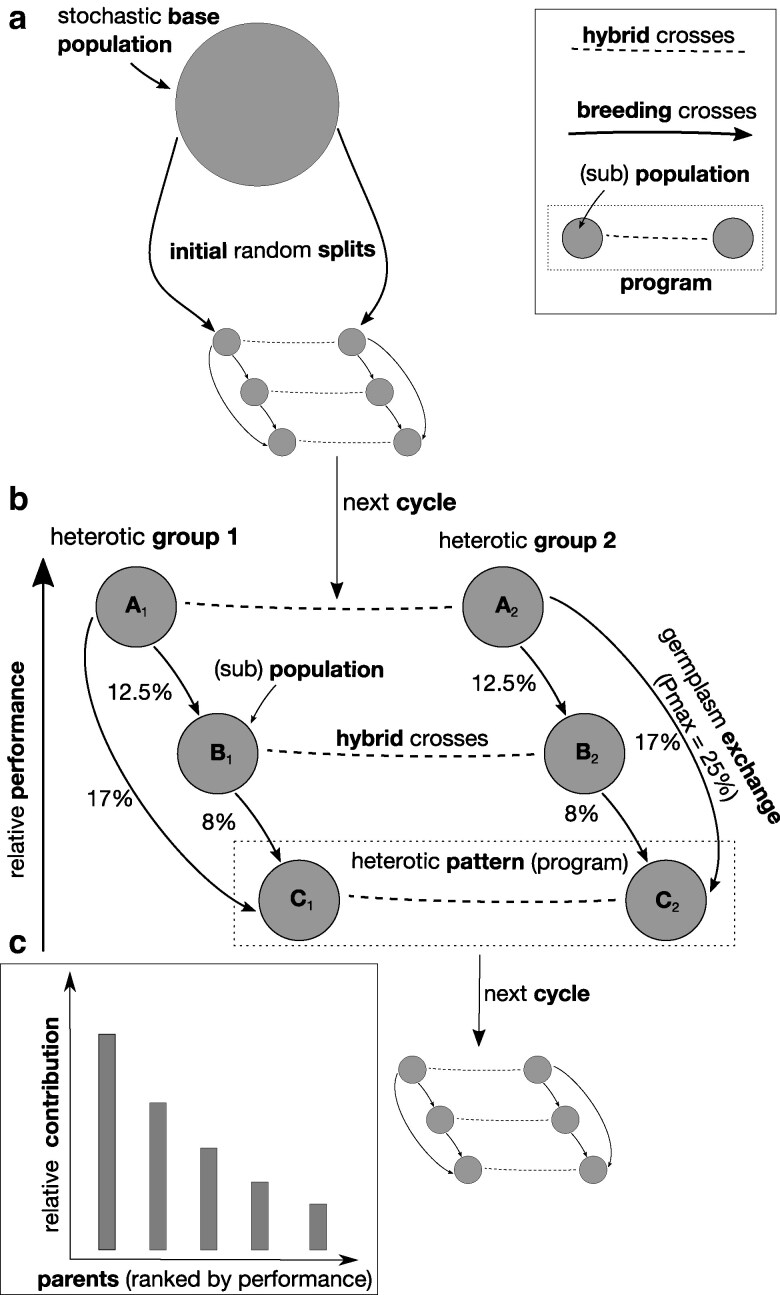
Schematic visualization of simulated hybrid breeding process using the distributed program structure as example. a) Stochastic creation of base population and separation into heterotic groups and subpopulations; b) detailed snapshot view of the processes happening within each cycle; c) relationship between the performance of selected breeding parents and their relative contribution to the next generation. The programs were ranked based on the average performance of their experimental hybrids in a given cycle. The percentage values next to the arrows connecting programs indicate what percent of the breeding crosses are conducted with lines from higher ranked program. The lower the performance rank of a program, the higher this percentage, with lowest ranked program receiving the maximum given by Pmax and the highest ranked program not conducting any crosses with outside line. The figure was reproduced with permission from Technow et al. (2021).

The simulation process followed closely the one in our previous work (Technow et al. 2021), which was designed to reflect the features of long-term hybrid breeding operations observed in practice (Duvick et al. 2004; Mikel and Dudley 2006). It is visualized in Fig. 4 for the most general *distributed* program structure, of which the others are special cases (more details on this and the other structures will be given below). On the outset, a base population comprising 1,000 inbred lines was generated stochastically with the approach described by Montana (2005). This was done in such a way that the expected linkage disequilibrium between two loci *t* Morgan apart equaled $r^{2}=0.5\cdot 2^{-t/0.1}$, and all minor allele frequencies were distributed uniformly between 0.35 and 0.50. This base populations was then separated at random into two equally sized heterotic groups (arbitrarily labeled “1” and “2”) and, depending on the scenario, further into subpopulations within those. Subheterotic patterns where then formed by pairing one subpopulation from one heterotic group with one subpopulation from the other group. We will subsequently refer to those pairs also as “breeding programs” or simply as “programs.” Hybrids were produced strictly by crossing lines across heterotic groups while breeding crosses to generate the next generation of recombinant lines where done strictly within the designated heterotic groups. However, while hybrid crosses were done only within a heterotic pattern (ie program), breeding crosses could be done within and among subpopulations, depending on the scenario (Fig. 4b).

#### Evaluation of genetic performance

The GCA of each line, the primary selection metric used in hybrid breeding (Reif et al. 2005), was evaluated with an incomplete, reciprocal mating design (Melchinger et al. 1987 ; Seye et al. 2020 ) by performing crosses with five random lines from the subpopulation of the opposite heterotic group of the same program. The performances of the resulting testcross hybrids were then obtained with the E(NK) model as previously described and averaged. The multienvironment field trials (MET) in which testcross evaluation is carried out necessarily are an imperfect representation of the full TPE. This was reflected here by sampling the ET weights used for calculating testcross hybrid performances from a Dirichlet distribution with concentration parameter equal to true ET frequencies in the TPE multiplied by 500. This was done separately for each program. Thus, the METs of the different programs differed slightly from each other and from the true TPE. A normally distributed noise variable with zero mean was then added to reflect residual noise. The variance of this noise variable was chosen in such a way that the resulting GCA values had a heritability of 0.75 on an entry mean basis, a value achievable for traits like yield in multienvironment, multitestcross trials (Schopp et al. 2015).

The so obtained “observed” GCA values were then also used to predict the performance of all possible intergroup hybrids within the program by summing the GCA values of the corresponding parent lines (Reif et al. 2005). The top hybrids were then selected based on these GCA predictions and their true performance determined according to the E(NK) model, using the true weights of the ET in the TPE. The size of this selected class, which represents the set of advanced experimental hybrids considered for commercial release, depended on the scenario and more details will be given below. The true performances of these experimental hybrids were then averaged and this average used to quantify the overall performance of the program in a given cycle for the purpose of ranking the programs for determining the proportion of breeding crosses with lines from other programs, as will also be described below in more detail. Commercial breeding programs typically release only very few hybrids from each cycle to farmers. The performance of the top experimental hybrid across all programs, ie the one that would be targeted for commercial release in practice, was consequently defined as the practically relevant performance metric for the whole breeding operation in that cycle and also used as a metric of genetic gain.

#### Selection of breeding crosses

To determine which lines contributed to the next generation through breeding crosses and by how much, they were assigned a usage probability that was the product of an individual and population level relative contribution value. The individual level contribution value was a function of the rank of a line within its subpopulation and determined as follows. First, the lines were ranked according to their observed GCA values (Fig. 4c). Low ranked lines received a contribution value of zero, which excluded them as potential breeding cross parents. The contribution values of the top ranked lines within each subpopulation were then drawn from a Dirichlet distribution. The concentration parameters of this distribution were chosen in such a way that the relative contributions halved with every one-fifth rank quantile. Thus, with, eg 25 lines selected as parents, the highest performing line contributed approximately twice as much to the next generation as the fifth ranked line and four times as much as the 10th ranked line. How many lines were selected depended on the scenario and will be detailed when describing the alternative breeding program structures. These individual contributions thus correspond to the *disproportional* contribution scenario as defined in Technow et al. (2021), and reflect the practical reality observed in long-running commercial breeding operations (Rasmusson and Phillips 1997; White et al. 2020).

The population level contribution values regulate how much the lines of one program contribute to the breeding crosses of another. The example visualized in Fig. 4b will be used for describing how they were derived. In each cycle, the programs (labeled “A,” “B,” and “C,” with subscript 1 or 2 indicating the heterotic group) are ranked according to the average performance of their experimental hybrids, as described above. Generally, the lower the performance rank of a program, the higher the proportion of breeding crosses with lines from other programs, from zero for the highest ranked program (A), which consequently does not use any outside lines in breeding crosses that cycle, to a value of Pmax for the lowest ranked program (C) and equidistantly spaced values for the program(s) with intermediate ranks (B). Furthermore, exchange of germplasm between programs happened strictly in the direction from higher to lower ranked programs. In the example as well as throughout this study, Pmax was 25%. Thus in the example, the highest ranked program A performs no crosses with external lines, the intermediately ranked program B uses lines from other programs in 12.5% of its new crosses and the lowest ranked program C uses lines from programs A and B in Pmax=25% of its crosses. The exact proportion derived from each of the higher ranked programs was proportional to how much their performance differed relatively. If, as in the example, the performance difference between programs C and A is twice as large as that between C and B, lines from program A will contribute twice as much to program C than lines from program B. Specifically, in this example, 17% from A and 8% from B for a total of 25%. Because the ranking and relative performances of the programs can change over cycles, those relative contributions are derived anew each time as well. As stated, use of lines for breeding crosses, within and across subpopulations, happened strictly within their respective heterotic groups, eg subpopulation ${\text{C}}_{1}$ could receive lines only from ${\text{A}}_{1}$ and ${\text{B}}_{1}$. This scheme reflects that in practice, successful programs have little incentive to use outside material for improving their genetic base while less successful programs will do so at increasing rates in order to close the observed performance gaps. To arrive at the final usage probabilities, the individual and population level contributions were then multiplied with each other. Then the breeding crosses were determined by sampling with replacement the lines proportionally to their usage probabilities. While this could result in multiple instances of the same cross (ie between high performing lines), selfings were excluded. From each of these crosses, one recombinant line was obtained through three generations of single seed descent selfing, followed by a final doubled haploidy generation to remove residual heterozygosity. These new recombinants then formed the next breeding cycle, fully replacing the previous generation. Generations were thus discrete and a line could be used as a breeding cross parent in only one cycle. The breeding simulation was run for 30 cycles and 350 independent repetitions were conducted for each of the scenarios studied. Unless specified otherwise, all results presented are the averages over these 350 replications.

### Alternative breeding program structures

Following Technow et al. (2021), we distinguished and explored three main alternative scenarios for how breeders might choose to structure their breeding programs. The already mentioned *distributed* structure (Fig. 4) is characterized by the presence of multiple smaller programs that interact with each other through the exchange of genetic material in the form of breeding cross parents, as described in the previous section. In the *centralized* structure only a single, large program exists, with the only separation being into heterotic groups, which is fundamental for hybrid breeding (Melchinger and Gumber 1998). The final structure considered in our previous work was the *isolated* structure (*iso*), which resembled the distributed structure, except that no exchange of genetic material across subpopulations took place (ie Pmax=0%). Here, we add two further structures where genetic exchange took place, but only sporadically. These could thus be considered as intermediates of the distributed and iso structures. In the *iso7* structure, exchange happened every seventh cycle and in the *iso21* structure only once in cycle 21. In the cycles in which exchange took place, it happened exactly as already described for the distributed structure. Note that in iso21, the cycle in which exchange took place immediately followed the TPE shift under the sudden TPE change scenario, as a dramatic environmental change might prompt breeders to break their isolation temporarily and seek out genetic material from programs that navigated the shift better. Before the exchange cycle, the iso and iso21 structures operate identically and no differences between them should be expected.

The distributed and isolated structures comprised five programs each with one subpopulation of size 100 per heterotic group. In every cycle, 25 lines were selected from each subpopulation as potential breeding cross parents. The number of experimental hybrids selected per program was 25. In the centralized structure, the number of lines per heterotic group was 500, with 125 selected as parents. Here, the number of experimental hybrids was 125. The total number of lines and selected breeding parents per heterotic group as well as experimental hybrids was with 500, 125, and 125, respectively, the same across all structures. Thus, the alternative structures can be considered as using approximately the same amount of resources in this regard. Because of the uneven use of parental lines in crosses and the possibility of them contributing to multiple programs, we quantified for each cycle the effective number of breeding cross parents contributing to the next generation with the previously defined Inverse Simpson Index, with b now being the vector of relative contributions (Boichard et al. 1997). This was done across the subpopulations within the arbitrarily chosen heterotic group 1. Averaged across cycles, the resulting values were 61.2 (distributed), 62.0 (centralized), 64.6 (iso7), 65.0 (iso21), and 65.1 (iso).

### Recorded metrics

Several metrics were used to study the short- and long-term behavior of the simulated breeding system.

(1) The performance value of the top hybrid identified in a given cycle from each structure was defined as the performance of this structure in that cycle.

(2) The proportion of GCA to total genetic variance (%GCA) is a key metric describing the amount of additive genetic variation exploitable for heritable genetic improvements, as well as for identification of superior hybrid combinations in a given cycle. It was calculated as described in Technow et al. (2021), except that we used the *MCMCglmm* R package (Hadfield 2010) to estimate variance components. %GCA was estimated separately for each program, for the structures where there were multiple, and then averaged to arrive at a single value per cycle.

(3) The correlation between GCA effects (GCA correlation) was used as a measure of transferability of genetic effects across programs. It was evaluated by performing testcrosses with partners across subheterotic patterns. For example, the lines from ${\text{A}}_{1}$ were testcrossed with lines from ${\text{B}}_{2}$ and the so obtained GCA values correlated with those from the regular testcrosses with ${\text{A}}_{2}$ (Fig. 4). For computational reasons, this was done for only one subheterotic pattern and only every third cycle.

(4) The global Fst statistic was used to quantify genetic differentiation between subpopulations in the distributed and isolated structures. Fst values between populations were calculated separately for each heterotic group and then averaged, ie they quantify differentiation within a heterotic group. The Fst statistics were calculated with function fs.dosage from the *hierfstat* package in R (Goudet and Jombart 2022).

(5) Following Technow et al. (2021), we used the proportion of loci with a minor alleles frequency below 5% ($U_{W}$) as a measure of allelic diversity. This measure also quantifies the thickness of the extreme tail of the allele frequency distribution. The higher it is, the more the distribution is “*U-shaped*,” which was identified as a key determinant for the amount of additive genetic variation observed in natural populations (Hill et al. 2008). This calculation was done separately for each subpopulation and then averaged to arrive at a single value per cycle for each.

(6) The effective population size ($N_{e}$), calculated according to the method described in Corbin et al. (2012) for estimating constant effective population size, was used as a holistic measure of the diversity and coverage of genetic space. It was calculated across the subpopulations within each heterotic group and then averaged.

(7) The ranking of subpopulations determines flow of genetic material and reflects how much each contributes to overall, commercially relevant, genetic gain (measured as performance of top performing hybrid in a given cycle). We therefore defined a metric called *top-rank stability* which represents the probability that the top-ranked program in cycle n−1 is also the top-ranked program in cycle *n*. The value for cycle *n* was obtained as the proportion of the replications of the simulation in which the top-ranked program in cycle *n* was the same as the one in cycle n−1. To aid interpretability, the resulting curve across cycles was smoothed using loess.

All computations were conducted in the R environment for statistical computing (R Core Team 2021).

## Results

### Absolute performance over time

For brevity, results will only be shown for *K* values of 1, 8, and 15, representing genetic additivity, and intermediate and high genetic complexity, respectively.

#### Gradual TPE change

**Fig. 5.**
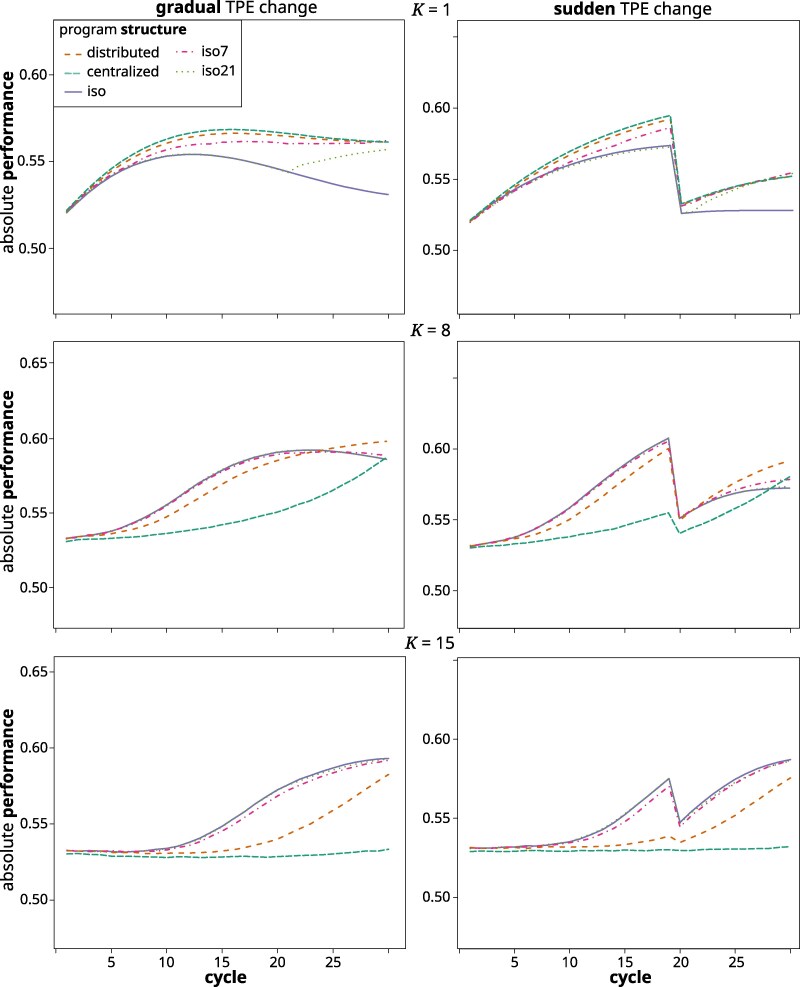
Absolute performance across cycles of the different breeding program structures in the gradual (left) and sudden (right) TPE change scenario at *K* levels of 1 (top), 8 (middle), and 15 (bottom)

At K=1, performance in all structures increased until about cycle 10 but then leveled off or even decreased, as particularly pronounced in the case of the iso structure (Fig. 5). During intermediate cycles, the centralized structure had the highest performance, followed by the distributed and iso7 structure. A noteworthy exception to the general trend of stalling or declining performance in later cycles was structure iso21, for which performance increased again noticeably after the germplasm exchange event in cycle 21. At K=8, the isolated structures reached a higher performance level quicker and were superior during the first 20 cycles, after which performance stalled and then dropped off toward the end. The distributed structure followed the isolated structures closely and replaced them as the superior structure by cycle 25 as performance kept increasing throughout. The centralized structure saw noticeable cycle over cycle genetic performance improvements only after cycle 15, after which performance improved at a faster rate than for the other structures. At K=15, none of the structures showed any performance improvement until cycle 10. From there on, performance increased for the isolated structures, with a slowing rate toward the end. The iso21 structure very closely followed the iso structure, as did iso7, albeit with a slightly lower rate of gain. Performance for the distributed structure started to make improvements after cycle 15, for the centralized structure it stayed virtually flat throughout.

#### Sudden TPE change

At K=1, performance increased for all structures from cycle 1 on, with the centralized structure reaching the highest level until the TPE shift, followed by the distributed and iso7 structures. Performance increase in the iso and iso21 structures was lowest. After the TPE change at the end of cycle 20, performance collapsed dramatically to close to the initial value for all structures. After this, it remained flat in the iso structure, whereas it started to recover again in all others and in a similar way. At K=8 and 15, a similar pattern as in the gradual scenario was observed, except that like for K=1, performance collapsed after the TPE change and then started to recover. In contrast to K=1, this recovery was also observed for the iso structure.

### Relative ranking of program structures over time

**Fig. 6.**
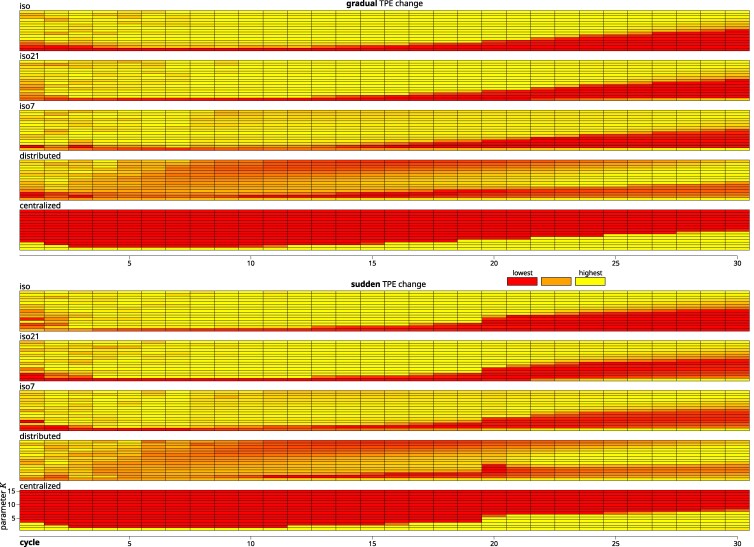
Relative performance ranking of structures across cycles, *K* levels and TPE scenarios. The brighter the color, the higher the relative performance of the structure compared to the others. Color values were linearly scaled to the range between the highest and lowest performing structure within this cycle, *K* and TPE combination. The values represented are the average performances across the replications of the simulation.

The relative performance of the different structures was visualized for all combinations at each combination of TPE scenario, *K* and cycle in Fig. 6. This figure represents the relative ranking of each structure with colors scaled to be situated linearly between the lowest and highest performing structure.

In both TPE change scenarios, the isolated structures (iso, iso7, and iso21) generally were the superior structures during the first 15 cycles, except under *K* values below 3 in the very first cycles and throughout for K=1, where the centralized structure was superior . The iso7 structure generally ranked very similarly to the iso structure, with the notable exception being K=1, where the iso structure was the lowest ranking throughout, while iso7 maintained an intermediate rank. Similarly for iso21 after the exchange cycle. During the later cycles, the isolated structures generally were superior only at higher levels of *K*. For intermediate values of *K*, the distributed structure was superior and for lower levels of *K* the centralized structure. The distributed structure throughout the cycles and across levels of *K* when not superior maintained at least an intermediate rank.

### Quantitative genetic parameters

The various quantitative and population genetic parameters used to measure system behavior showed similar general patterns across both TPE change scenarios. For sake of brevity, we will therefore illustrate these only for the gradual scenario. We will also highlight results only for *K* levels of 1, 8, and 15. Full results for all levels of *K* and both TPE change scenarios are available as supplement (Files S1 and S2).

#### %GCA

**Fig. 7.**
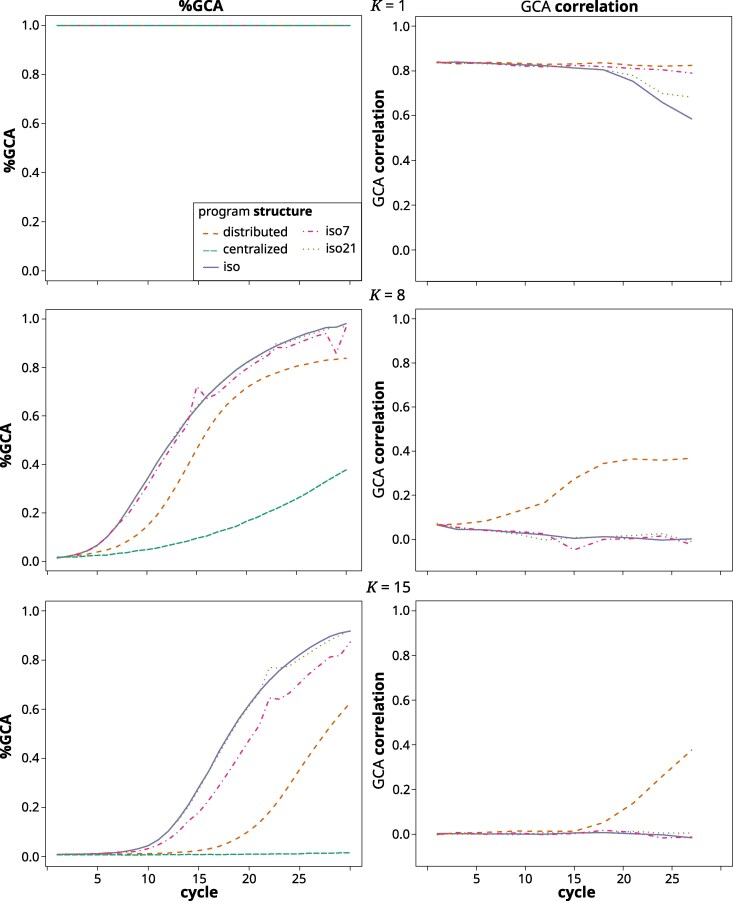
%GCA (left) and GCA correlation (right) across cycles for the different breeding program structures in the gradual TPE change scenario at *K* values of 1 (top), 8 (middle), and 15 (bottom). The values represented are the averages across the replications of the simulation. Note that the GCA correlation does not apply to the centralized structure.

For K=1, %GCA was equal to 100% for all structures, as expected (Fig. 7). At K=8 %GCA started from just above zero and then increased from there. This happened fastest in the iso and iso21 scenario, which reached above 50% before cycle 15 and close to 100% by the final cycle. The iso7 structure followed slightly behind the other two. It also showed a sudden small increase after the second and third germplasm exchange cycle and a sudden decrease after the final exchange cycle 28. iso21 also showed a small increase after its exchange cycle. All of these were short lived and the effect dissipated the following cycle. %GCA increased slower in the distributed structure, which reached 50% only by cycle 15 and just above 80% by the final cycle. The increase was slowest in the centralized structure, which had barely reached 40% by the final cycle. These trends were similar but more pronounced at K=15, where the iso7 structure now fell noticeably behind the iso structure, the distributed structure reached 50% only around cycle 25 and the centralized structure remained at close to zero throughout.

#### GCA correlation

At K=1, the GCA correlation throughout remained at close to 0.85 for the distributed and iso7 structures (Fig. 7). For the iso and iso21 structures, it started falling after cycle 15, with a stronger decline in the iso than the iso21 structure. At K=8, the correlation was below 0.1 initially and declined further toward zero for the iso, iso7, and iso21 structures. In case of the distributed structure, the correlation increased until it reached a plateau of just below 0.4 after cycle 20. At K=15, the correlation remained at zero for all structures, except the distributed structure, where it started to increase from cycle 15 onward and reached close to 0.4 by cycle 27.

#### Fst

**Fig. 8.**
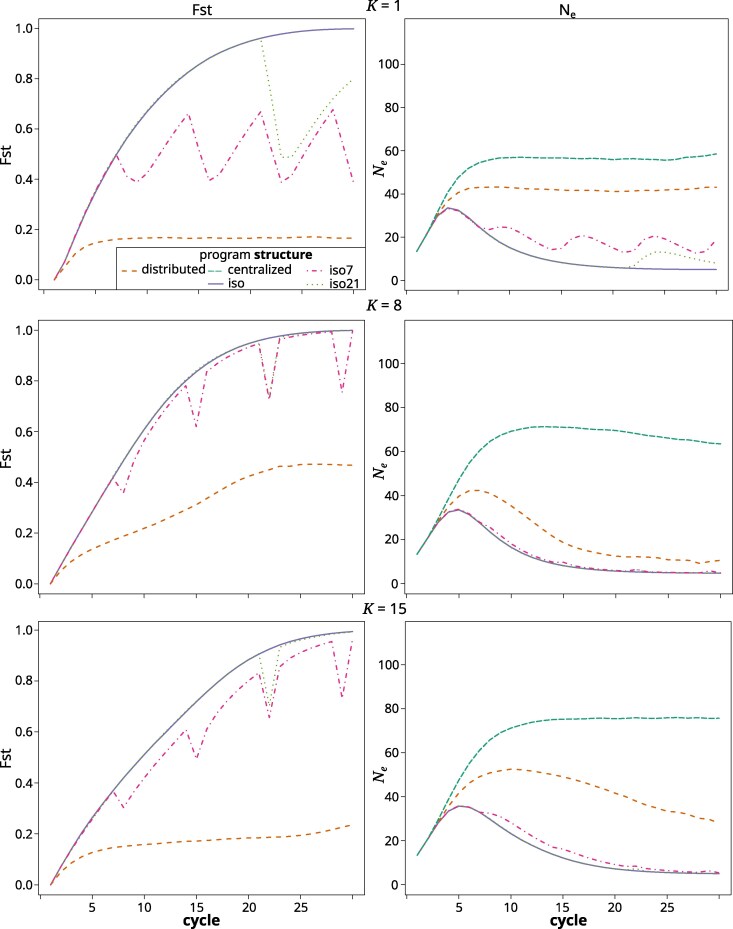
Fst (left) and $N_{e}$ (right) across cycles for the different breeding program structures in the gradual TPE change scenario at *K* values of 1 (top), 8 (middle), and 15 (bottom). The values represented are the averages across the replications of the simulation. Note that the Fst among programs is not applicable to the centralized structure.

At K=1, Fst among subpopulations in the distributed structure quickly increased to a value of 0.17 and then remained there for the remainder (Fig. 8). In the iso structure, it quickly rose to above 0.6 by cycle 10 and ultimately reached 1.0 in the final cycles. The iso7 and iso21 structures shared the high rate of Fst increase. While Fst dropped after each exchange cycle, it rose again immediately thereafter. For the iso7 structure that led to Fst oscillating around a value of 0.5 from cycle 7 onward. At K=8, Fst in the iso structure again rose quickly and reached 0.5 before cycle 10. The iso7 and iso21 structures again showed drops in Fst after germplasm exchanges. In contrast to K=1, the Fst value prior to the exchange was regained after just two cycles. Fst in the distributed structure rose more slowly than in the other structures and remained at around 0.47 from cycle 23 onward. The trends at K=15 were similar to those at K=8, except that the Fst increased at a slower rate in all structures, values in iso7 were now noticeably lower than in iso and iso21 and in the distributed structure they did not reach a plateau and were lower overall.

#### Effective population size

At K=1, $N_{e}$ across subpopulations in the centralized and distributed structures rose from the initial value and quickly stabilized at around 55 and 42, respectively (Fig. 8). In the different iso, iso7, and iso21 structures, $N_{e}$ rose initially until cycle 5 but then dropped again. In the iso and iso21 structures, it converged toward a value of 5, which is the number of subpopulations within each heterotic group. After each exchange cycle in the iso7 and iso21 structures, $N_{e}$ increased briefly but then dropped again. This led to an oscillating pattern around a value of 17 in iso7. At K=8, the centralized structure again had the highest $N_{e}$, which reached above 70 at the peak around cycle 10 before slowly decreasing again. In contrast to K=1, $N_{e}$ of the distributed structure initially increased to above 40 but then started falling again and ultimately stabilized at around 10. The pattern for the iso, iso7, and iso21 structures was similar to K=1, except that the effect of the germplasm exchanges on $N_{e}$ was barely noticeable. The pattern at K=15 was similar to K=8, except that the decrease in $N_{e}$ after the early peak in the distributed and isolated structures was slower. In the distributed structure, $N_{e}$ reached above 50 at its peak and was still above 25 in the final cycle. In the three isolated structures, it nevertheless had declined toward 5 toward the end.

#### U-shapedness

**Fig. 9.**
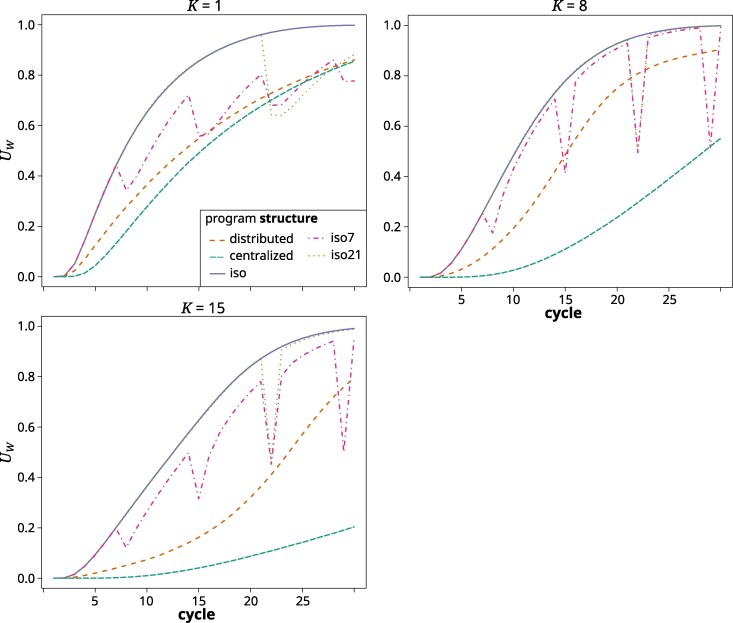
Proportion of loci with MAF <0.05 (U-shapedness, $U_{W}$) across cycles for the different breeding program structures in the gradual TPE change scenario at *K* values of 1 (top left), 8 (top right), and 15 (bottom left). The values represented are the averages across the replications of the simulation.

The proportion of loci with minor allele frequency (MAF) <0.05 within subpopulations ($U_{W}$) increased across cycles and program structures and levels of *K* (Fig. 9). The increase was always strongest in the iso structure, where it reached a value of close to 100% in the final cycles. At K=1, it increased with a similar overall rate and reached a similar final value in all other structures. The iso7 and iso21 structures showed a drop after each exchange cycle, followed by a renewed increase. At higher complexity levels, this drop after the germplasm exchanges was even more pronounced but also was reversed quickly in the following cycle. For the distributed and centralized structures, the increase was slower at higher *K* values, strongly so for the latter, where $U_{W}$ reached just about 20% by the final cycles.

#### Top-rank stability

**Fig. 10.**
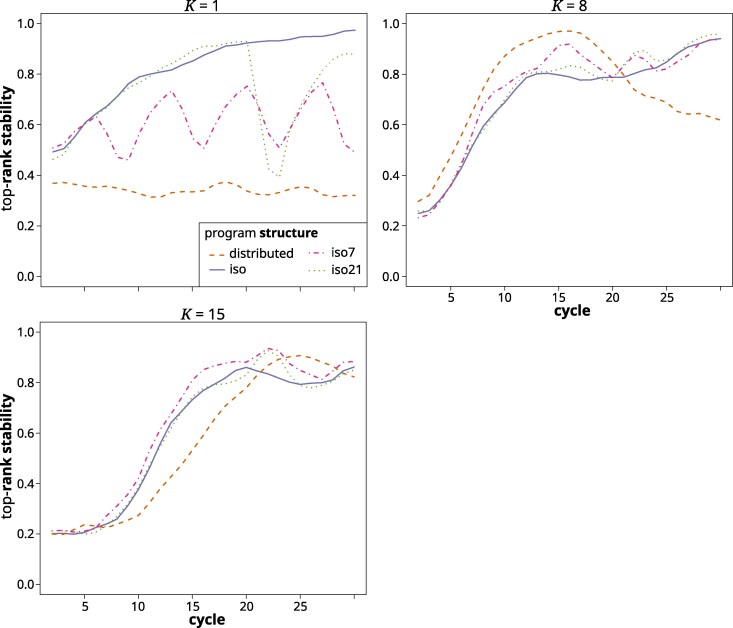
Top-rank stability (probability that the top-ranked program in cycle *n* was the same as the one in cycle n−1) across cycles for the different breeding program structures in the gradual TPE change scenario at *K* values of 1 (top left), 8 (top right), and 15 (bottom left). Note that this metric is not applicable to the centralized structure.

At low *K*, the top rank stability was highest in the iso structure, where it increased over time to close to 100% and lowest in the distributed structure, where it remained constant at just below 40% (Fig. 10). In the iso7 and iso21 structures, the value dropped after each exchange cycle, followed by a relatively rapid increase to the prior level. At intermediate and high values of *K*, it increased from around 20%, the value indicating randomness, to 75% and higher in all structures. The exception was the distributed structure at intermediate *K*, where it quickly rose to close to 100% by cycle 15 but then dropped again to 60%. In the sudden TPE change scenario, the top-rank stability for all structures dropped noticeably after the environmental shift, but then quickly regained its prior trajectory (File S2).

## Discussion

Additive genetic variation, a prerequisite for heritable selection gains, is given only in the simplest gene to phenotype model of purely additive gene action. As a model of biology this seems inadequate on the basis of scientific advances in the field of biology demonstrating that quantitative traits are the product of complex interactions at the molecular, metabolic, and physiological level (eg Carlborg and Haley 2004; Phillips 2008; Saha et al. 2011; Wilkins et al. 2016; Boyle et al. 2017; Fiévet et al. 2018; Vasseur et al. 2019). Infinitesimal and purely additive gene action also seems inadequate to explain why commercial plant breeding operations have maintained impressive levels of genetic gain, despite structures not conducive for this success under this model (Rasmusson and Phillips 1997; Duvick et al. 2004; Technow et al. 2021). Under all other models of genetic complexity, additive genetic variation is an emergent property of the nonlinear gene networks underlying phenotypic variation. It has to be “exposed” by constraining genetic variability through fixation or near fixation of genes and gene complexes (Wade 2002; Cooper et al. 2005; Hill et al. 2008). A process that leads to the effective linearization of these networks.

In Technow et al. (2021), we investigated how genetic complexity affects the performance and properties of different hybrid breeding program structures. There we found that as genetic complexity increases, increasing constraints on the accessible genetic variability, eg by population stratification, are required for short- and long-term genetic gain. Here, we explored this further by adding the dimension of environmental complexity, in the form of a gradually or suddenly changing TPE.

### Balance between short- and long-term selection response

In Technow et al. (2021), we evaluated the relative superiority of the alternative structures in terms of their performance in the final cycle of the breeding simulations. Because the TPE was static, the final performance value reflected how well a structure traversed the genetic landscape in search for higher local optima or even the global one.

With a dynamic TPE, however, the genetic landscape changes as well (Figs. 1, 2, and 3). The local optima achieved in the final cycle are thus not indicative of the local optima identified in earlier cycles and hence how well and quickly a given breeding structure was able to react to historical shifts in the genetic landscape. For reference, even with deployment of all currently available breeding technologies like doubled haploids (Dwivedi et al. 2015) and whole genome selection (Meuwissen et al. 2001), each breeding cycle, from initial cross to the point where the next generation of proven, elite lines are recycled, will require three to five calendar years (Heffner et al. 2010). The 30 cycles covered in this simulation thus easily span a century and with that the potential for massive shifts in the biotic and abiotic environment, management practices, and market demands (Collins and Chenu 2021; Zhao et al. 2022; Shi et al. 2025). We therefore assessed the balance of short to medium and long-term selection response under a shifting genetic landscape, by evaluating whether the different structures were able to both maneuver and achieve genetic gain within the current genetic landscape while maintaining the ability to maneuver also in future landscapes.

In general, we found that during the earlier cycles (1 to 10), the isolated structures delivered superior rates of performance improvement, followed by the distributed structure and with the centralized structure faring poorest. For the medium- and long-term, the isolated structures retained their superiority only at high degrees of complexity. This was because the loss of genetic variability (measured by $N_{e}$ and $U_{W}$) in the isolated structured became severely limiting, while at the same time the other structures constrained their genetic space enough to reach a %GCA sufficient for effective selection response. Thus, during the last third of the simulation, the centralized structure was superior for lower values of *K* and the distributed structure for intermediate to high complexity levels.

The pattern observed in the final cycles reflects our previous findings (Technow et al. 2021), except that the relative performance of the structures in regards to *K* was shifted to higher values. For example, while in Technow et al. (2021), we found the centralized structure to be superior only until K=4, it was so now until K=7. Similarly, the distributed structure there was superior between K=5 to 8 and here from about 8 to 13. This is because of the added dynamics of a shifting TPE, which increase the importance of preserving genetic variability to react to the future environmental shifts. The isolated structures struggled with this. For lower to immediate values of *K*, where genetic variability had been largely exhausted by the final cycles (Fig. 9), the isolated structures even experienced a decrease in their absolute performance level (Fig. 5). This was because previously advantageous allele and gene complexes that had become fixed in earlier cycles, later became neutral or detrimental for performance. There is thus a balance between short- and long-term selection response, which translates to the compromise between exposing and exploiting additive genetic variation within the current TPE and retaining genetic variability for adapting to the future TPE. In other words, GxE and a shifting TPE expand the complexity of the potential state space (ie the potentially relevant and accessible genotype configurations). They also create greater uncertainty associated with pursuing any specific selection trajectory. This confers an advantage for more landscape exploration before committing to move toward a particular local optimum. The different breeding program structures adjust the balance of exploration and exploitation differently. The distributed structure, which maintains separate subprograms with constant exchange between them, strikes a middle ground between the centralized and isolated structures. It combined the desired features of both while mitigating some of their drawbacks. For example, it was able to quickly generate enough additive variation, measured as %GCA, to facilitate almost immediate selection response (Fig. 7), but without the dramatic drop in genetic variability (Fig. 9) and effective population size (Fig. 8) experienced by the isolated structures. Across a wide range of degrees of complexity, this allowed it to quickly generate cycle over cycle performance improvements during the early cycles, while maintaining these responses also in the later cycles. As outlined in Technow et al. (2021), the distributed structure closely reflects the breeding program model that emerged organically over the last century within large scale hybrid breeding operations (Crabb 1947; Cooper et al. 2014).

The notable exception to these trends is the special case of K=1, where all variation is inherently additive. The outlined effects of population subdivision on the emergence of additive variation consequently do not apply. Here, the centralized structure, with its substantially higher $N_{e}$, was superior across all cycles, as would be expected from well-established results pertaining to the assumptions of the infinitesimal model with additive effects (Barton et al. 2017).

### Restoring genetic variability through germplasm exchange

Restoring the genetic variability of isolated public breeding programs through occasional germplasm exchange was suggested to maintain their long-term adaptability and competitiveness (Fonseca et al. 2021). We evaluated this option in the form of the iso7 and iso21 structures that allowed for sporadic germplasm exchange between the otherwise isolated programs. Interestingly, these closely followed the performance development of the completely isolated iso structure. The exchange thus had little to no effect on the medium- to long-term behavior of the evolutionary trajectory. As can be seen for $U_{W}$ (Fig. 9), and, albeit less clearly, for $N_{e}$ (Fig. 8), the sporadic germplasm exchange did have a short-term effect on genetic variability. This, however, dissipated very rapidly in a matter of a cycle or two. The explanation can be found in the near zero across program correlation of GCA effects in the isolated structures (Fig. 7). With near zero GCA correlation, germplasm that is elite in one subgroup is not likely to be also elite in the other and hence will quickly be purged. As we pointed out here and previously (Technow et al. 2021), not just the (relative) amount of additive variation, but also the directionality and magnitude of additive effects are dependent on the germplasm context. Thus, the additive effect of a gene or gene complex in one context is not necessarily predictive of its (additive) effect in another. With the distributed structure, this GCA correlation started increasing as soon as a sizable amount of GCA variation was exposed. Owing to the constant exchange of germplasm, the subprograms thus were converging toward a section of the genetic landscape within which additive genetic effects remained transferable across genetic backgrounds. Also of note is that for the distributed structure, the rank-stability metric in the final third of the breeding simulation was around 0.6, ie low enough to indicate frequent reranking of subprograms. During the final phase of the simulation, where access to sources of genetic variability was most crucial, the direction of the flow of germplasm hence changed relatively frequently. Interestingly, both the maintenance of transferability of gene effects, as well as the fluidity of the ranking of the programs, existed despite considerable subpopulation differentiation (Fig. 8). The different subpopulations thus did not occupy an entirely similar genetic space, meaning that this structure indeed exhibited the features of a distributed search strategy in complex genetic space (Podlich and Cooper 1999). The relatively high Fst values are also indicative of the presence of significant amounts of latent genetic variability at the metapopulation level, ie across the ensemble of subprograms comprising the breeding structure (Wade and Goodnight 1998). This is even more the case for the isolated structures in which the very high Fst values indicated fixation or near fixation of contrasting gene complexes across the different subpopulations. However, because of the described difficulties with germplasm exchange, this latent genetic variability was not readily accessible.

The problem of incorporating unadapted and inferior germplasm for maintaining genetic variability has long been recognized and several strategies to mitigate undesirable effects on the recipient germplasm exist (Allier et al. 2020). Here, we highlight that this problem does not just apply to exotic or ancient genetic resources derived from, eg gene banks (Yu et al. 2016) or landraces (Böhm et al. 2017). It also applies to sources that can be considered “elite” within their own genetic context and adapted to the same TPE. Usually, it is recommended to adapt these genetic resources before their integration with the elite germplasm pool during several cycles of “prebreeding.” This can be done rapidly and cost effectively with the help of genomic selection (Polzer et al. 2025). Because of the context dependency of genetic effects, it is, however, important that this adaptation is done within the genetic context of the recipient germplasm (Fonseca et al. 2021). Even more conservative strategies are to focus exchange on narrow genomic regions with depleted variability or the introgression of specific haplotypes with demonstrated effects on target traits. The latter strategy seems most promising for addressing specific weaknesses of the elite pool, such as disease resistances, rather than for improving performance as such. But even then, the transferability of the identified effects from the external to the elite background has to be established (Mayer et al. 2020). In our simulation, the amount of breeding crosses with external germplasm was limited to a maximum of 25%. The persistence and impact of the exchanged germplasm would have been greater at higher exchange rates, because then the germplasm context of the recipient program after the recombination round would have been more similar to that of the donor program. In the extreme case, one could envision a strategy where the germplasm of the inferior programs is essentially replaced with that of the superior programs. This, however, is not common practice, as the resulting homogenization would largely defeat the purpose of maintaining separate breeding programs. In our simulations, genetic exchange happened strictly from higher to lower performing programs and only utilized their highest performing parental lines. These are reasonable default choices for practical breeding operations but limit the potential amount of external genetic variability available to the individual programs. The effect of relaxing those assumptions and exploring optimal germplasm exchange strategies under differing genetic and environmental complexity scenarios should be studied further.

The rapid genetic variability loss in the isolated structures could also have been mitigated by employing variability preserving selection techniques, such as optimal mate selection (Cowling et al. 2017) or techniques aiming to preserve haplotype diversity (Müller et al. 2018). De novo generation of genetic variability via DNA mutation (Durand et al. 2010) or epigenetic phenomena (Hauben et al. 2009) likely also contributes to the maintenance of long-term selection response in isolated breeding populations (Rasmusson and Phillips 1997; Dudley and Lambert 2010; Durand et al. 2010, 2015). While not considered in this study, complex mutations (Trujillo et al. 2022) and epigenetics (Bull 2014) can be represented within the *NK* modeling framework.

### Adaptation to environmental complexity and change

We considered two contrasting environmental change scenarios: gradual change, where the TPE shifts slowly from one end of the environmental spectrum to the next, and sudden change, where a dramatic shift happens from one cycle to the next. Overall, we found that the trends in relative superiority of the different structures across cycles and complexity levels as well as for most metrics used to describe these trends, were very similar in principle across both scenarios. The similar curves of lagged genotypic correlation between cycles (Fig. 1) point to a possible explanation: Over the long term, the TPE in both scenarios traverses a similarly wide range of ET, while in the short term, there is little practical difference between the directional change in TPE weights in the gradual scenario and their random fluctuations in the sudden scenario.

A sudden shift was expected to represent a shock to the system that can create bigger short-term disturbances, like throwing a large rock into water, than the effects of the gradual change, which is more like changing the tide. In our simulation, however, this shift happened only after cycle 20, after which the genetic space had already linearized to a large degree. The emerged additivity thus persisted regardless, eg there was no noticeable drop in %GCA after the TPE shift (Fig. 7). The structures thus quickly regained their selection trajectories and the dynamics prior to the shift continued. In other words, while the relative magnitudes and signs of the emerged additive gene effects might have changed, the system remained linearized. Its baseline topology (the *N* and *K* parameters) and more importantly the effective topology, ie the number of still segregating loci within each interactive pathway, were not affected. However, environmental shifts can directly alter genetic and phenotypic expression through increases in mutation rates (Carja et al. 2014) or induction of epigenetic changes (Turner 2009). Such shifts can even modify the topology of biological networks through recruitment of dormant pathways or increases in their interconnectivity (Iwasaki et al. 2013). While not considered here, such phenomena could be readily incorporated into the *NK* framework (Altenberg 1994).

It thus seemed that what matters most for the short- and long-term behavior of breeding germplasm, is whether or not the TPE changes, rather than how. As detailed above, the salient consequence of a shifting TPE is the increased importance of conserving genetic variability to facilitate adapting to future environmental conditions. In a static TPE and hence static genetic landscape, a local optimum identified early persists. There is thus less penalty for quickly and severely constraining genetic space as under a shifting TPE and landscape, where a local optimum can disappear over time. Thus, whereas under a static TPE, an exhaustion of genetic variability will lead only to a slowing rate of performance improvements, under a dynamic TPE it can mean a decline in absolute performance level, as observed for the isolated structures (Fig. 5). Regardless of whether the long-term environmental shift occurs gradually or suddenly, environmental conditions continuously change and fluctuate across all time scales (Bernhardt et al. 2020). This was represented in our simulations by small random cycle over cycle variations in TPE weights. These short-term fluctuations might in fact help to conserve genetic variability (Bell 2010; Abdul-Rahman et al. 2021), because the small and nondirectional changes in the underlying genetic landscape continuously perturb selection trajectories and thus prevent or slow down the premature fixation of gene complexes that might become disadvantageous later.

The gradual and sudden TPE change scenarios were chosen to represent the opposing poles of the environmental change spectrum (Alley et al. 2003). The E(NK) framework allows modeling of various other scenarios. For example, the occurrence of extreme events (Heinen et al. 2025) or an increase in the variance of environmental conditions (Lawson et al. 2015). However, as these scenarios also imply a changing TPE and hence selection target, we expect similar behavior as in the scenarios studied here. We also briefly evaluated a cyclical change scenario, where the TPE oscillates across the environmental spectrum (Bernhardt et al. 2020). Also here we found the same general trends (results not shown).

The threat of climate change and the renewed appreciation for the role of GxE have led to an increased urgency in the plant breeding community to enable germplasm to adapt to future environmental challenges. This has also led to an awareness of the need to align the MET in which the germplasm is evaluated to the on-farm TPE it ultimately has to perform in (Cooper et al. 2022). This has not always been consensus. Plant breeders historically often preferred the increased precision and convenience of a fixed network of well-managed, homogeneous and high yielding locations, over trialing under the more realistic but also more challenging conditions encountered on-farm (Ceccarelli and Grando 1996; Bänziger and Cooper 2001). Our simulation assumed that the MET represented the different ET with a frequency close those in the TPE. A deepened appreciation for the need of constructing appropriate METs (Cooper et al. 2022) makes this not unrealistic. For example, with increasingly popular sparse experimental designs (Jarquin et al. 2020), the number of locations in a MET can be expanded within a fixed budget. Remaining distributional biases between MET and TPE can be mitigated by reweighing of observed ET frequencies (Podlich et al. 1999; Chenu 2015).

Beyond simply following the trajectory of the TPE, future environmental shifts can be anticipated by representing them in the MET. This can be done either by adjusting observed ET frequencies, evaluating and selecting the germplasm in managed stress environments (Bänziger and Cooper 2001) or virtually through predictive models (Technow et al. 2015; Messina et al. 2018). An even more explicit approach is selecting for designed ideotypes with physiological features expected to convey adaptation to the anticipated conditions (Semenov and Stratonovitch 2013; Hammer et al. 2020). Evaluating today’s germplasm under hypothetical future conditions can be valuable for identifying weaknesses that could critically limit adaptability to these conditions. However, as these efforts require resources, care should be taken that the *adjacent possible* (Kauffman 2014) performance under current and near-term conditions does not become an afterthought. Not least because of the inherent uncertainty involved in anticipating environmental conditions decades into the future. We made the simplifying assumption that environmental changes occur entirely by extraneous factors. This ignores the possibility of a bidirectional relationship between crop evolution and changes in environment and agronomic practices (Sadras and Hayman 2024). The coevolution of crops and weed, insect, and fungal species is an example of this (Kareiva 1999). In contrast to the natural environment, changes in agronomic management are largely directed and controllable. While beyond the scope of this study, the synergistic coevolution between crop genetics and agronomic practices (Evans 1997; Fischer 2009) is a fascinating idea worth of further exploration. Extensions of the *NK* model allow incorporating such bidirectional relationships (Suzuki and Arita 2005; Swanack et al. 2008) in the framework developed here.

We already highlighted the distributed structure as achieving the best balance between short- and long-term genetic gain. This structure can also enable adaptation to a wide array of possible scenarios under a changing TPE with uncertain future conditions and increased geographic heterogeneity (Rivas and Gonzalo 2025). Namely by not just distributing the germplasm under evaluation but also the environments under which it is evaluated. This in fact has been common practice in breeding programs in large organizations, where rarely two programs target entirely overlapping environmental zones. The diverging selection environments will reinforce the implications of a stratified germplasm space under complex trait genetics. For the isolated structures, it means a more severe genetic divergence and thus make the highlighted issues with germplasm exchange even more challenging. For a distributed structure with regular exchange, however, it could have the positive effect of retaining more (latent) genetic variability on the metapopulation level. It could also lead to exploration of more unique physiological adaptation strategies. A distributed germplasm strategy can thus maintain agrobiodiversity and thereby the resilience of agricultural production systems in the face of climate change (Ceccarelli and Grando 2022). And it can do so without compromising the overall performance level. The importance of local adaptation and optimization of crops from the perspective of farmers was also discussed by Cooper et al. (2022). An novel concept explicitly combining a distributed germplasm strategy with environmental stratification through participatory on-farm field experimentation was recently proposed by Santamarina et al. (2025) for the purpose of developing locally adapted crop mixtures.

### A framework for studying the short- and long-term behavior of breeding programs

As in our previous work, our primary objective was not to provide specific and ultimate answers as to the optimal design of breeding programs but to suggest a quantitative framework with which concrete questions can be studied. This framework comprises of a model of biological complexity and breeding as well as metrics for describing their behavior. The chosen model of complexity is based on the E(NK) model (Cooper and Podlich 2002), which is an extension of the *NK* model. The latter was developed by the theoretical biologist and complexity researcher Stuart A. Kauffman (Kauffman 1993) to study evolution in complex biological systems. *NK*, respectively E(NK), models are an attractive choice because they allow to generate genetic landscapes with finely tunable degrees of complexity. From the special case of K=1 representing intrinsic and complete additivity to almost intractable complexity at values of K=15 and beyond (Fig. 3). Another advantage is that *NK* models are computationally efficient, even at genome scale levels of dimensionality and interconnectedness. Finally, the *NK* model has established itself as a representative model of complex systems beyond biology. It can thus provide a canonical framework across a wide array of disciplines, including innovation management (Ganco 2017), physics (Qu et al. 2002), infrastructure design (Grove and Baumann 2012), and economics (Faggini and Parziale 2016).

A limitation of the generic nature of the E(NK) framework is that it allows only to address conceptual “what if” questions. For practical plant breeding operations E(NK) like models that reflect the features and topology of their actual germplasm space and TPE would be of great interest. For model organisms with simple genomes, it is theoretically possible to directly estimate topological features of *NK* like fitness landscapes through studying the effects of individual mutations on phenotypes. From those predictions of, eg accessible evolutionary trajectories can then be made (de Visser and Krug 2014). For agricultural crop species, with complex genomes and exposure to TPE landscape characterized by complex GxE interactions, this direct approach is infeasible. A more practical approach is to use biological models which do not attempt to directly link genome features to phenotypic variation. They instead represent, on the basis of prior biological experimentation, simpler, and intermediate hierarchical levels in the gene to phenotype map (Hammer et al. 2006, 2019). A prime example in an agricultural context are dynamic crop growth models. They represent the biological knowledge gained from decades of research on the interplay between plant physiology, soil science, and micrometeorology (eg Keating et al. 2003; van Ittersum et al. 2003). With the help of Bayesian hierarchical whole genome regression techniques (Technow et al. 2015; Messina et al. 2018; Powell et al. 2021), these models have successfully been applied to model germplasm-specific biological complexity at the scale and resolution required by practical plant breeding operations (eg Cooper et al. 2016; Onogi 2020; Diepenbrock et al. 2022; Jighly et al. 2023). This approach has also been called “BioWGP.” On the opposite end of the gene to phenotype map exist gene regulatory network models. These also have been applied in an agricultural and plant breeding context (Dong et al. 2012; Leong et al. 2025). They represent the complex relationships between genes, transcription factors, and environmental signals that regulate gene expression and are constructed from a combination of experimentally obtained “omics” data and prior information (Muhammad et al. 2017; Badia-i Mompel et al. 2023). The structure and properties of gene regulatory networks have been conceptualized in the *omnigenic* model (Boyle et al. 2017), which postulates that the polygenic nature of most quantitative traits is a consequence of effect propagation through these highly interconnected networks. Recently, Ružičková et al. (2024) developed a statistical approach that allows to apply this omnigenic model directly to empirical data sets. Their *quantitative omnigenic model* could be used to obtain germplasm specific estimates of allele effects within the general interactive network structure, similarly as the BioWGP approach. It thereby could generate E(NK)-like models of biological complexity tailored to a specific germplasm context. Crop growth models, gene regulatory networks, and other biological models, such as metabolic networks (Fiévet et al. 2010), used individually or combined in dynamic gene to phenotype systems (Kauffman 2004; Messina et al. 2025), could thus be deployed to reduce to practice the conceptual insights gained from the E(NK) framework. Conceptually, such models could capture the system topology, level of complexity, and emergent behavior underlying an existing germplasm and TPE. Optimization of long-term strategies for concrete breeding operations under historical or predicted future TPE scenarios is then conceivable.

### Simple solutions to very complex problems?

The most basic result from our work is that the optimal breeding strategy for balancing short- and long-term genetic gain depends almost entirely on the level of biological complexity inherent in the system. In particular, we want to emphasize that our and other’s results show that germplasm trajectories and behavior for the special case of pure additivity (K=1) can be dramatically different from even low to intermediate levels of nonadditivity (Rasmusson and Phillips 1997; Technow et al. 2021; Tessele et al. 2026). Biological complexity is an undeniable reality, even if it does not always express itself in the form of statistically detectable nonadditive variation (Huang and Mackay 2016). Current attention to optimizing genetic gain and managing genetic variation for the long-term is mostly based implicitly on K=1 additive and stationary infinitesimal type models, within an often static TPE. This does not adequately consider the details of selection trajectories the breeding program has to navigate under a combination of genetic complexity and a shifting environment.

In an opinion paper, renowned plant breeding scientist Major M. Goodman (Goodman 2002) linked the recurrence of so called plant breeding “bandwagons” (Simmonds 1991; Bernardo 2016), ie technologies and approaches that promise simple solutions for complex problems, to an underappreciation of biological complexity in the form of GxE interaction and epistasis. We believe that novel technologies can play an important role in helping plant breeders tackle the challenges of future climates and population demands. However, their effective use requires a framework to appropriately represent the biological complexity and emergent phenomena of the genetic and environmental space in which they hope to intervene. Such a framework must also be able to represent the self-organizing principles with which germplasm has successfully navigated challenging and complex GxE landscapes over the last century, and if structured and managed appropriately, will do so also in the future.

## Supplementary Material

jkag044_Supplementary_Data

## Data Availability

The authors affirm that all data necessary for confirming the conclusions of this article are represented fully within the article and its figures and supplemental material. The computer code for the simulations is provided as supplementary material (File S3). Supplemental material available at G3 online.
