# Supplementary material for: Back to the future 2: the implications of germplasm structure on the balance between short- and long-term genetic gain in a changing target population of environments
Source: G3 (Bethesda). 2026 Feb 19;16(4):jkag044. doi: 10.1093/g3journal/jkag044 (PMC13042289; doi:10.1093/g3journal/jkag044)
Supplement: jkag044_Supplementary_Data [file jkag044_supplementary_data.zip › File_S1_G3-2025-406286.pdf]

k = 1

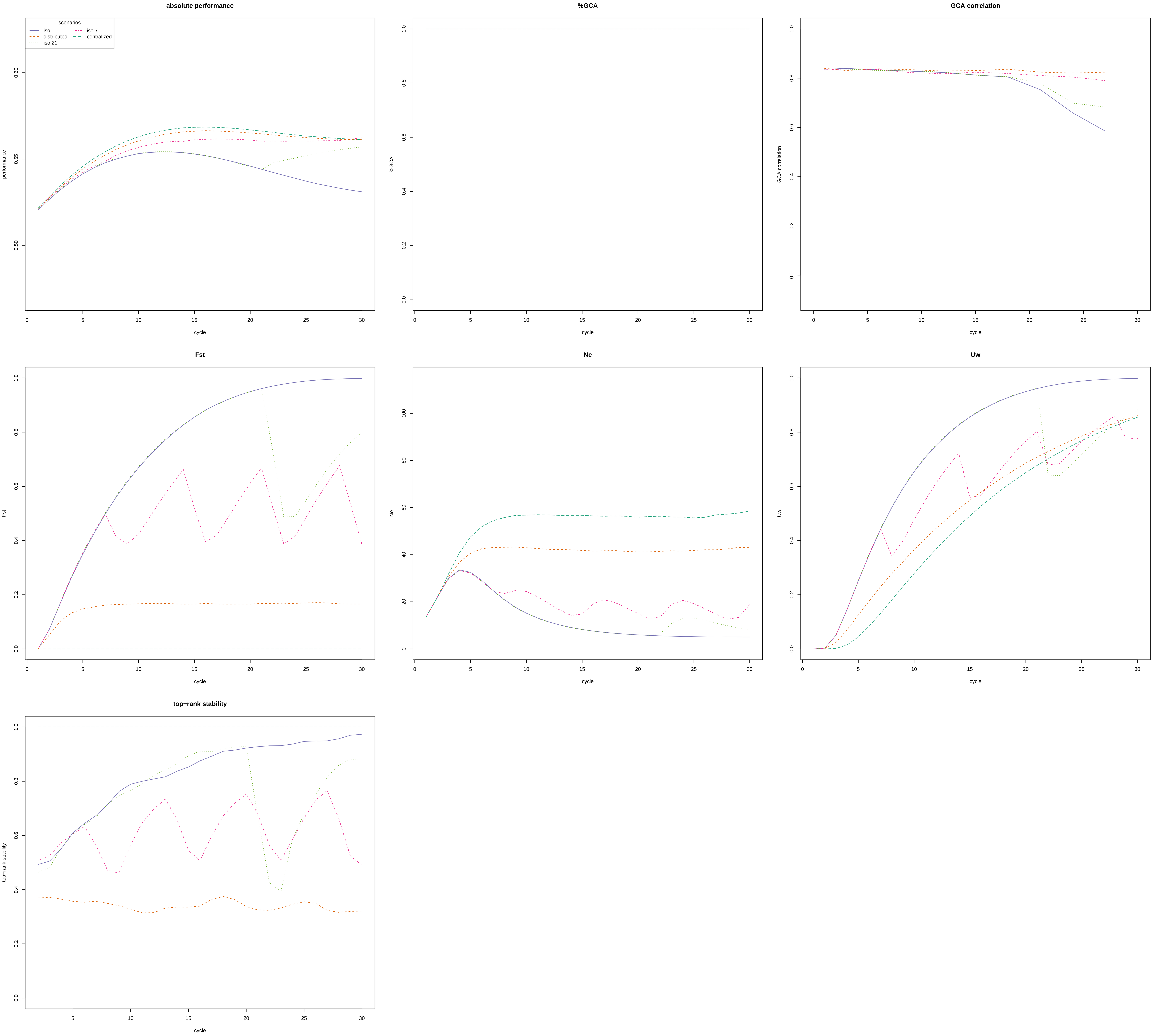
absolute performance
scenarios
iso
distributed
iso 21
iso 7
centralized
performance
cycle
%GCA
GCA correlation
Fst
Ne
Uw
top−rank stability

k = 2

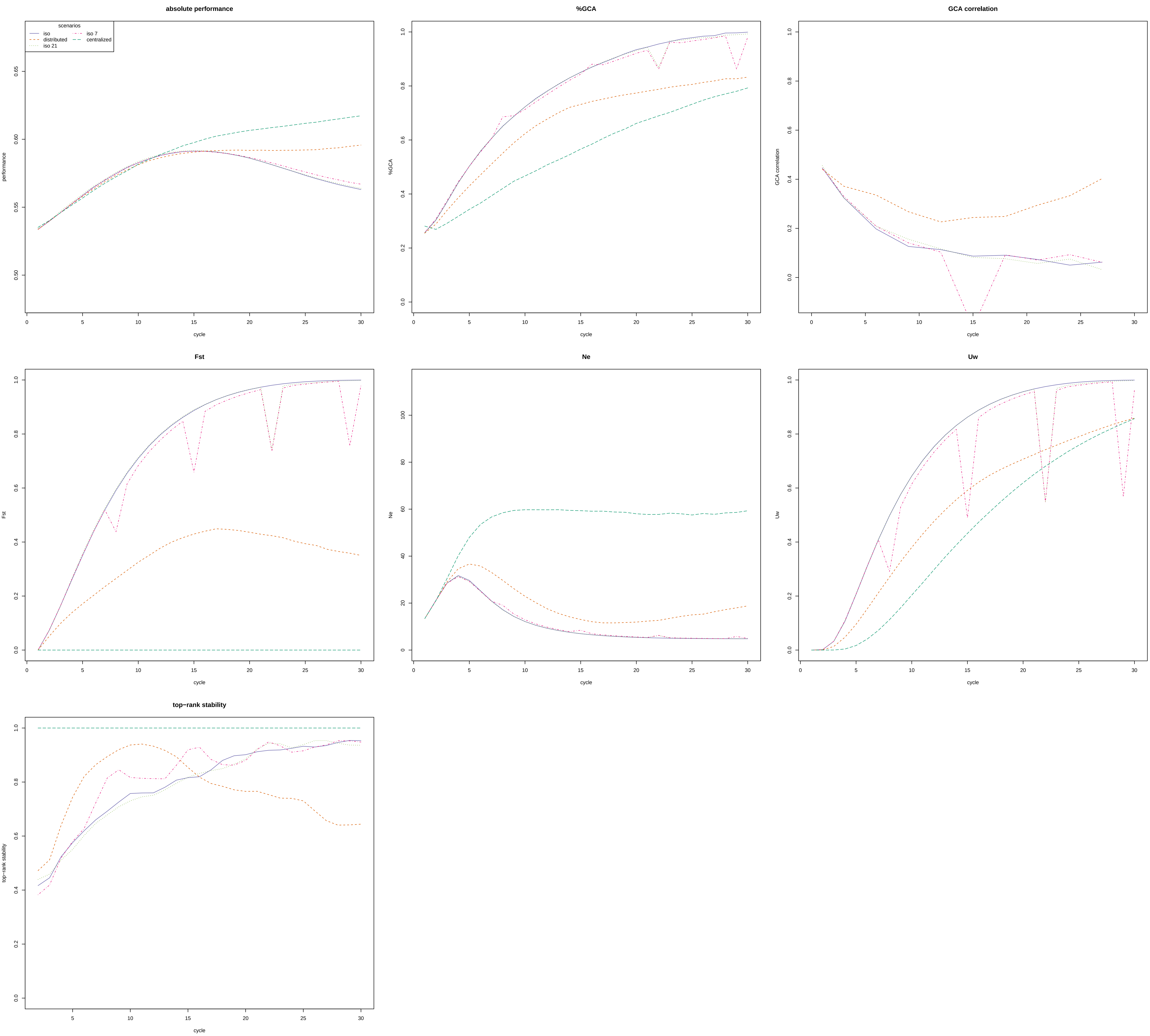
absolute performance
scenarios
iso
distributed
iso 21
iso 7
centralized
performance
cycle
%GCA
GCA correlation
Fst
Ne
Uw
top−rank stability

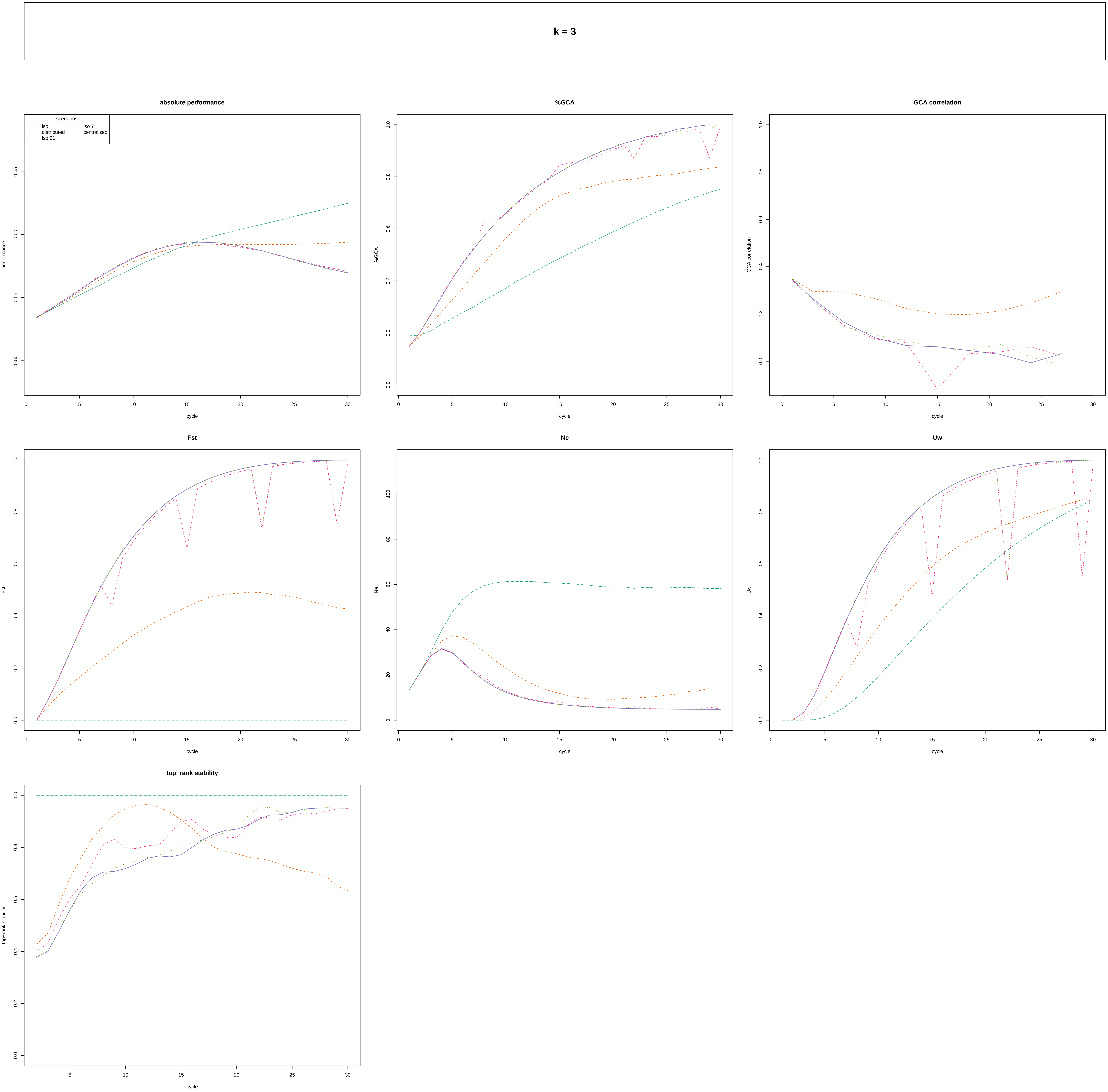
k = 3
absolute performance
scenarios
iso
distributed
iso 21
iso 7
centralized
performance
cycle
%GCA
GCA correlation
Fst
Ne
Uw
top−rank stability

k = 4

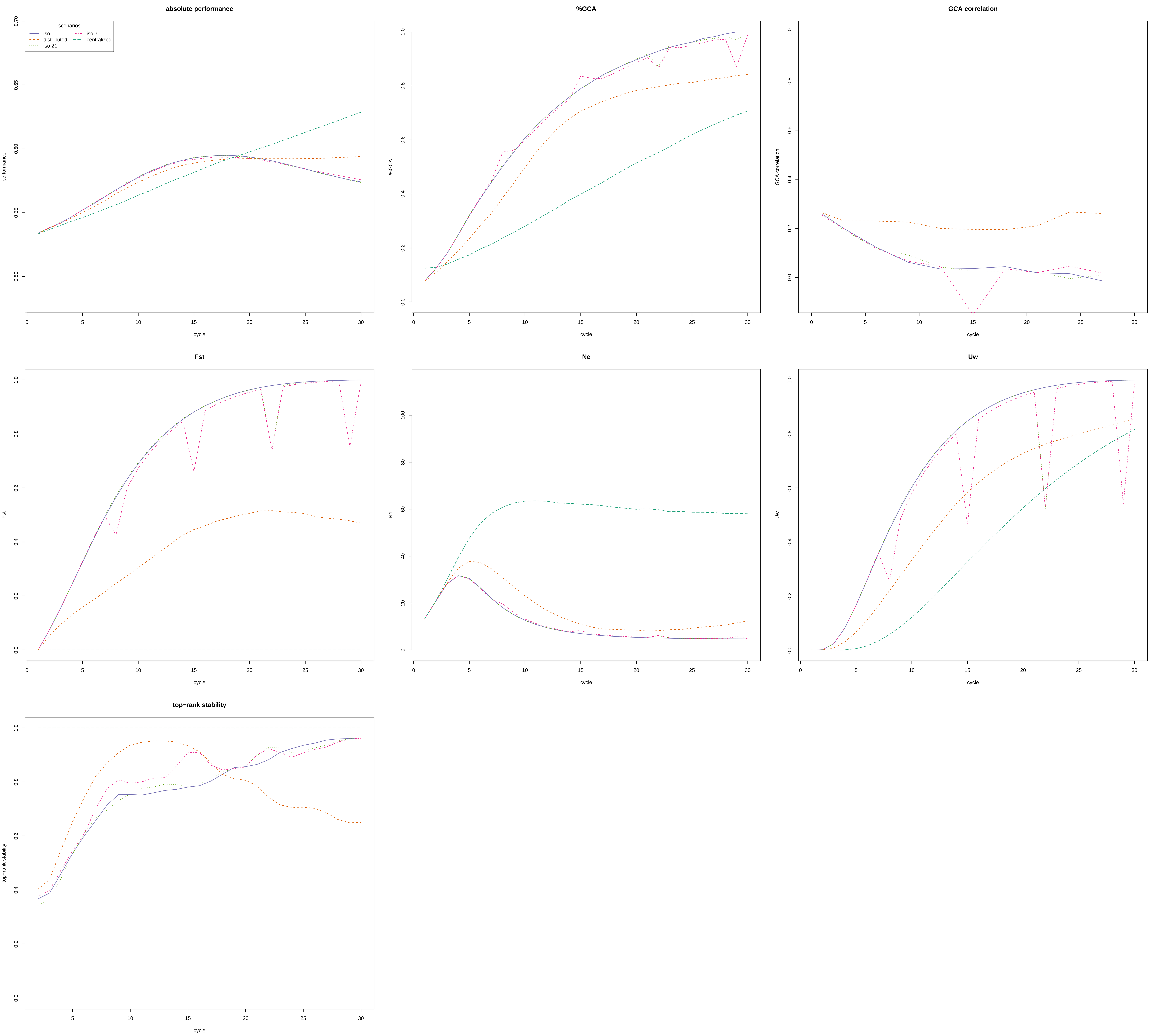

absolute performance
scenarios
iso
distributed
iso 21
iso 7
centralized
performance
cycle
%GCA
GCA correlation
Fst
Ne
Uw
top−rank stability

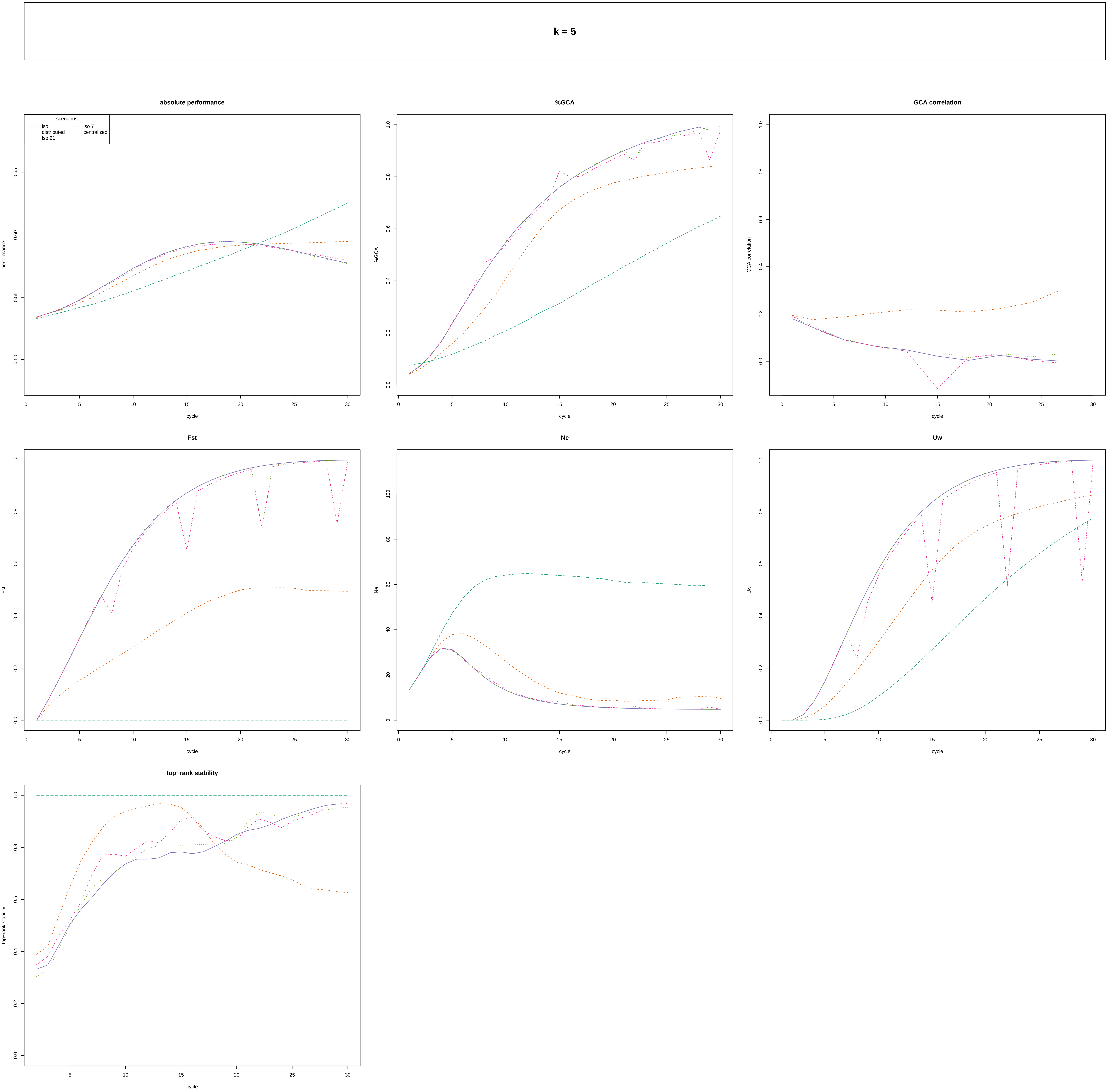
k = 5
absolute performance
scenarios
iso
distributed
iso 21
iso 7
centralized
performance
cycle
%GCA
GCA correlation
Fst
Ne
Uw
top−rank stability

k = 6

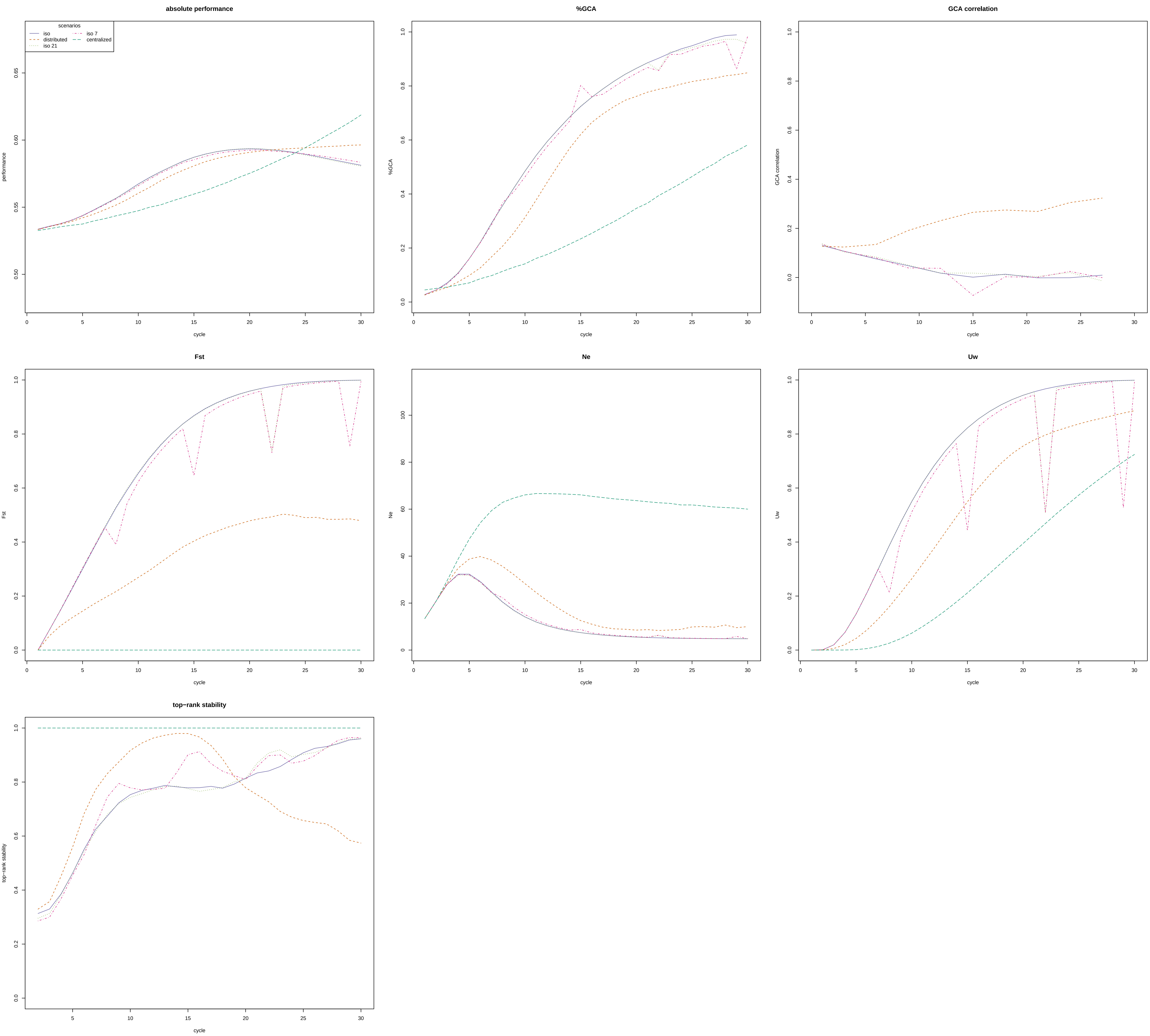
absolute performance
scenarios
iso
distributed
iso 21
iso 7
centralized
performance
cycle
%GCA
GCA correlation
Fst
Ne
Uw
top−rank stability

k = 7

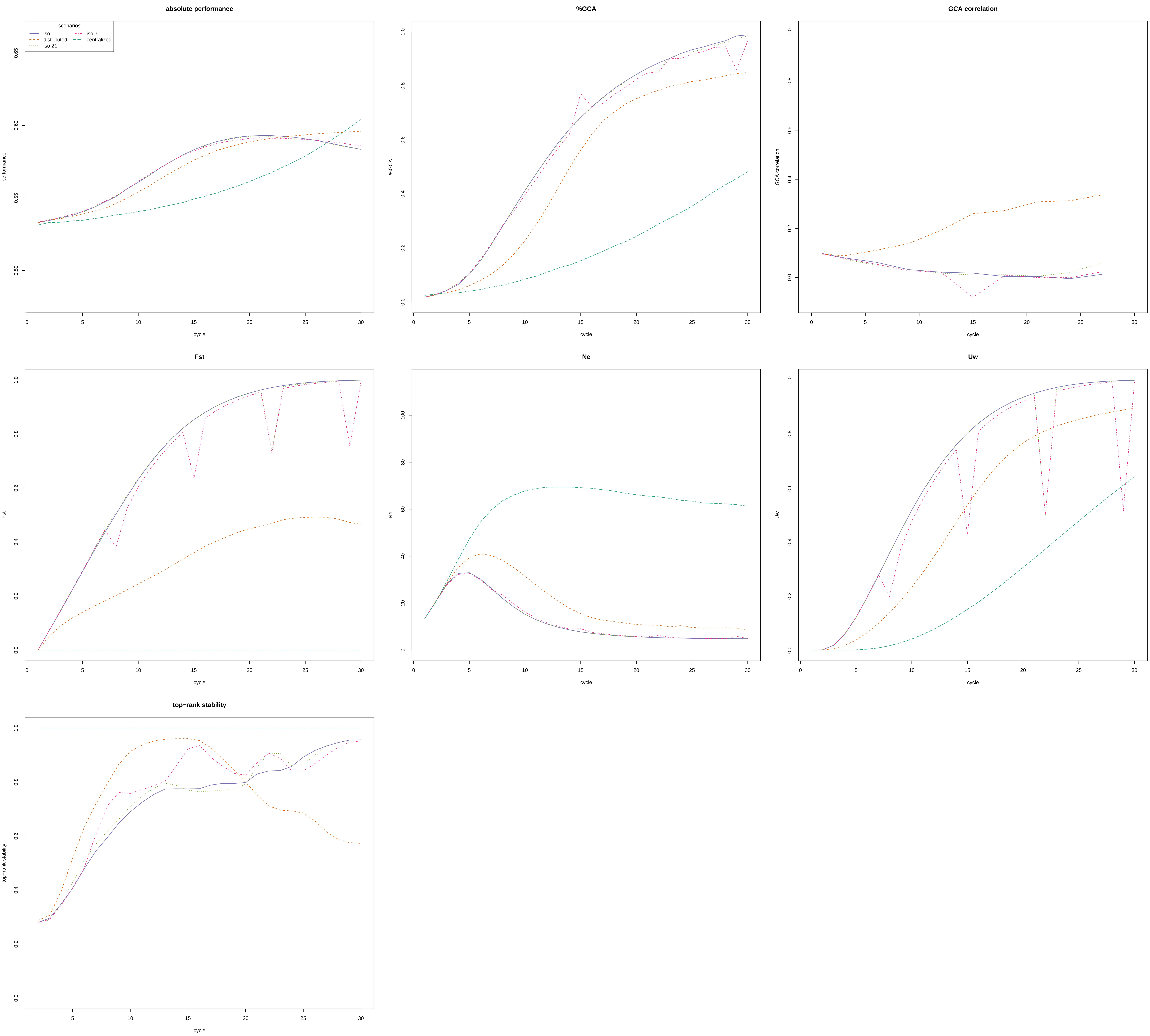
absolute performance
scenarios
iso
distributed
iso 21
iso 7
centralized
performance
cycle
%GCA
GCA correlation
Fst
Ne
Uw
top−rank stability

k = 8

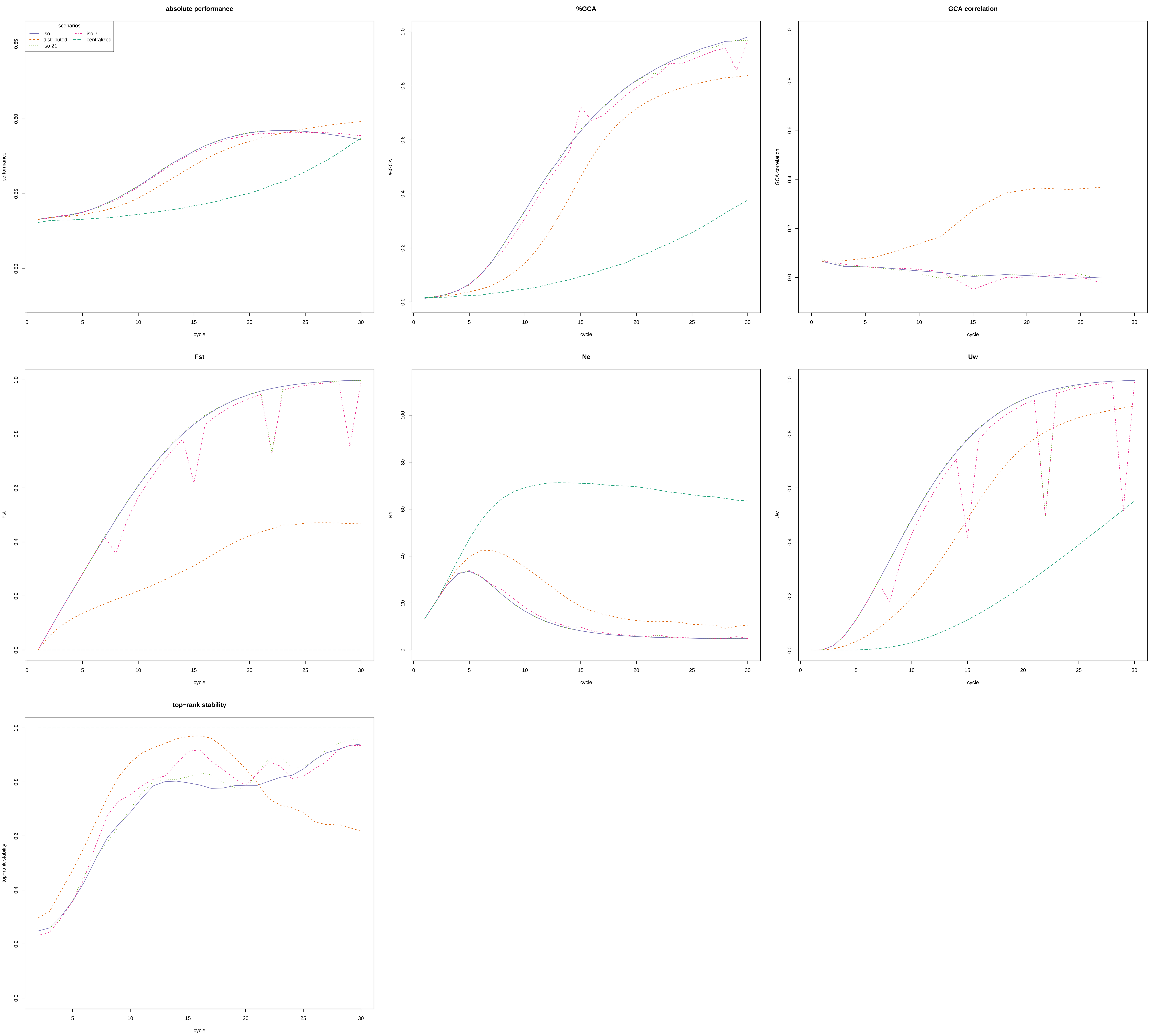
absolute performance
scenarios
iso
distributed
iso 21
iso 7
centralized
performance
cycle
%GCA
GCA correlation
Fst
Ne
Uw
top−rank stability

k = 9

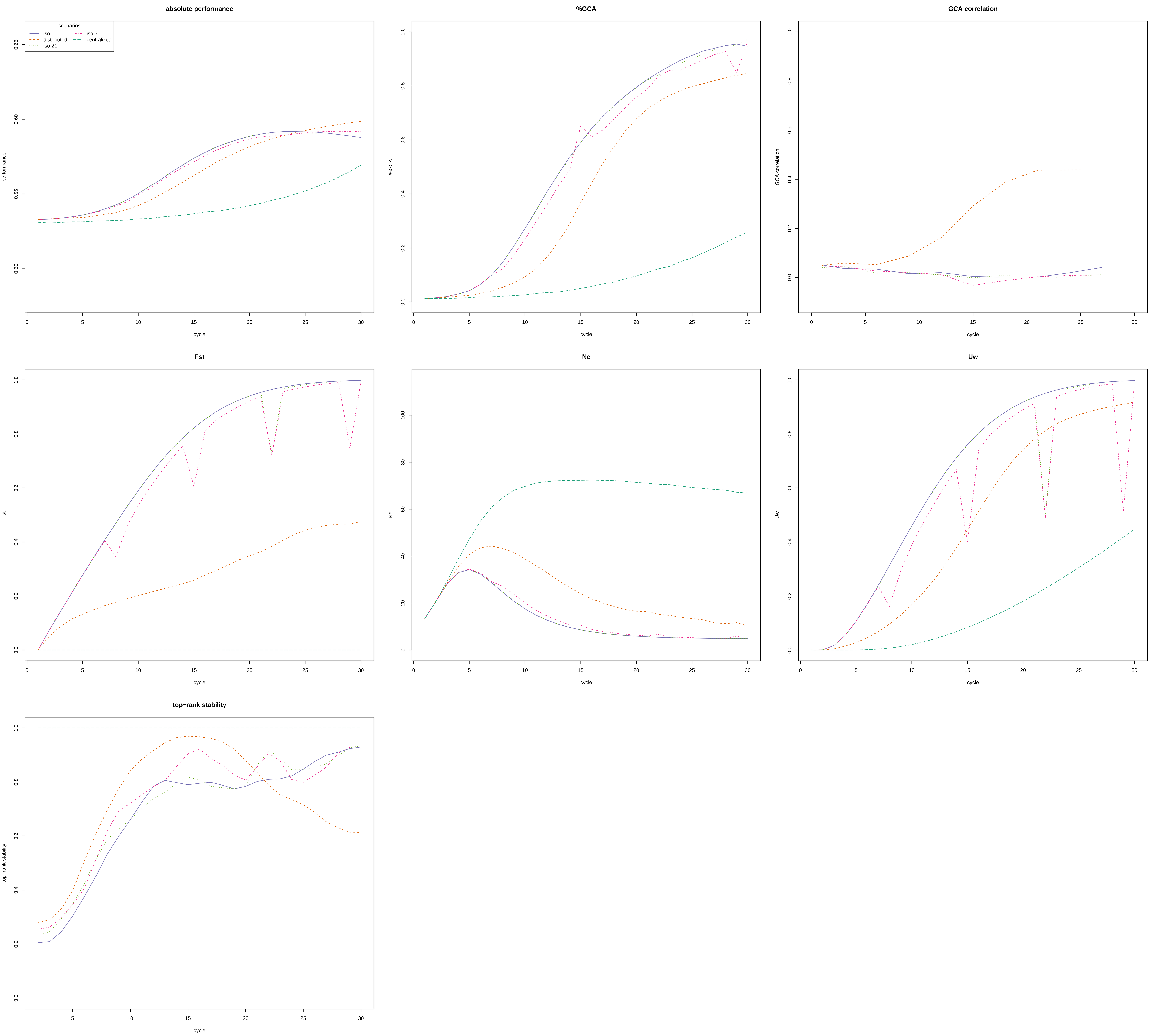
absolute performance
scenarios
iso
distributed
iso 21
iso 7
centralized
performance
cycle
%GCA
cycle
GCA correlation
GCA correlation
cycle
Fst
Fst
cycle
Ne
Ne
cycle
Uw
Uw
cycle
top−rank stability
top−rank stability
cycle

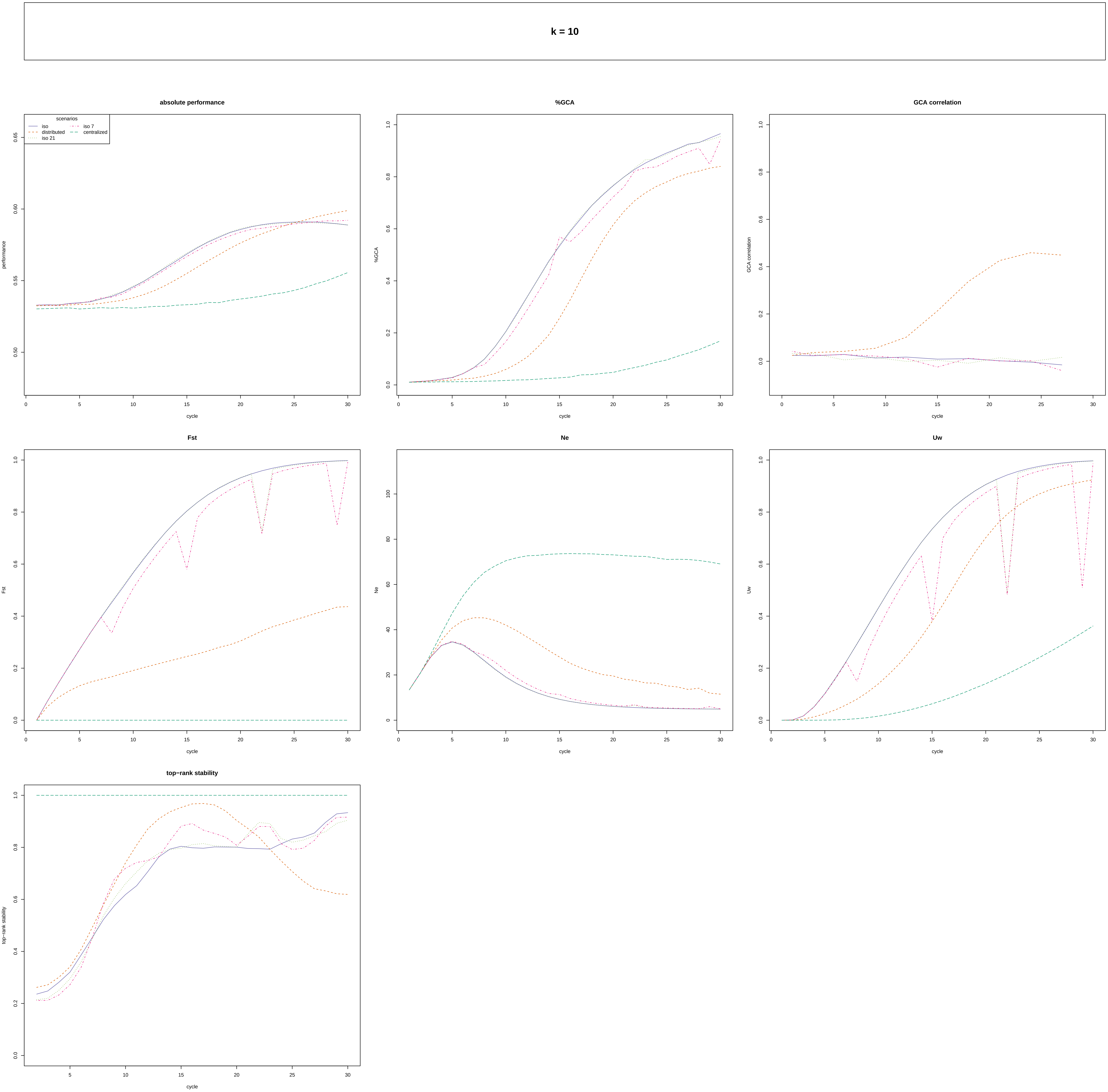
k = 10
absolute performance
scenarios
iso
distributed
iso 21
iso 7
centralized
performance
cycle
%GCA
GCA correlation
Fst
Ne
Uw
top−rank stability

k = 11

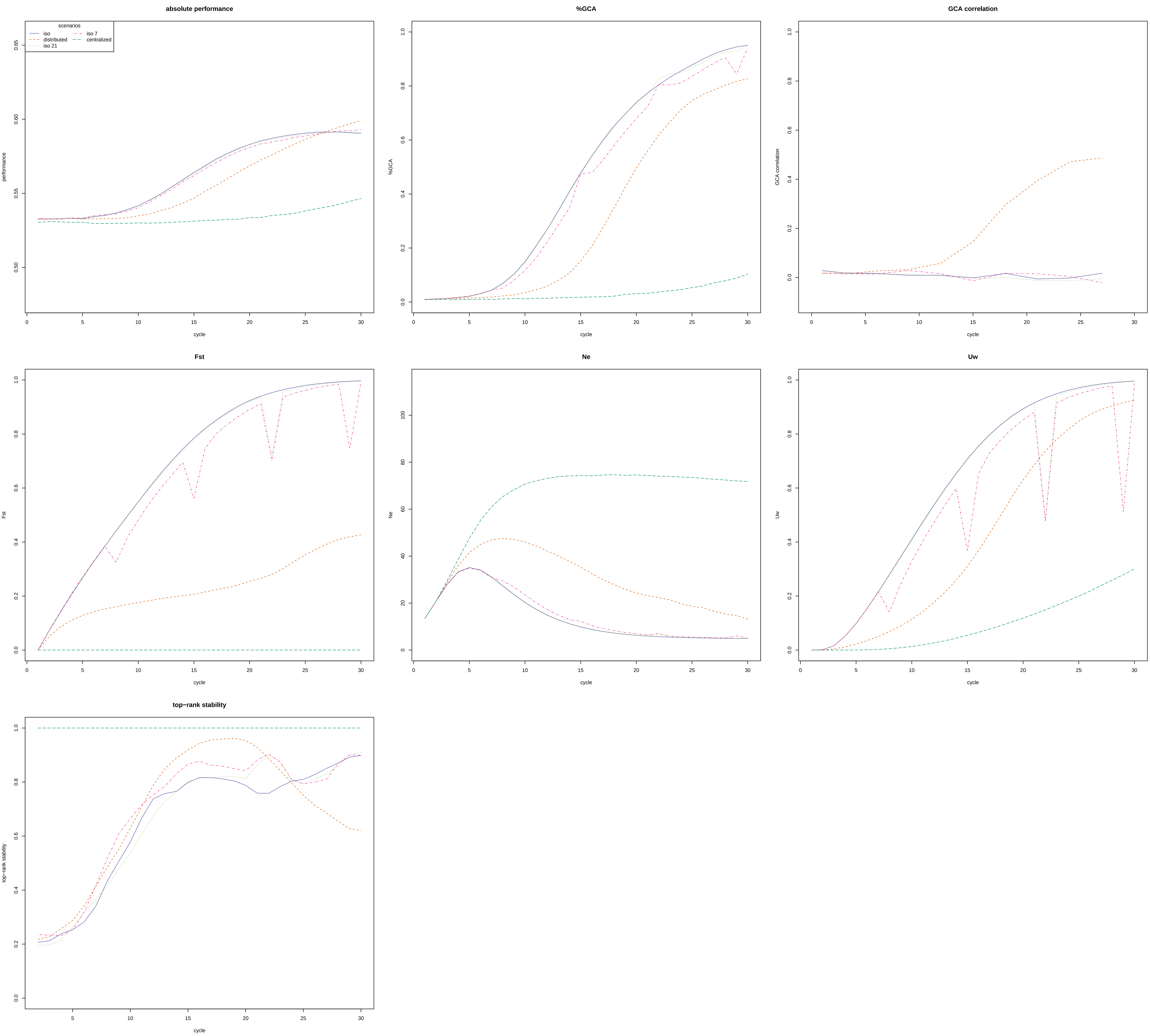
absolute performance
performance
cycle
scenarios
iso
distributed
iso 21
iso 7
centralized
%GCA
GCA correlation
Fst
Ne
Uw
top−rank stability

k = 12

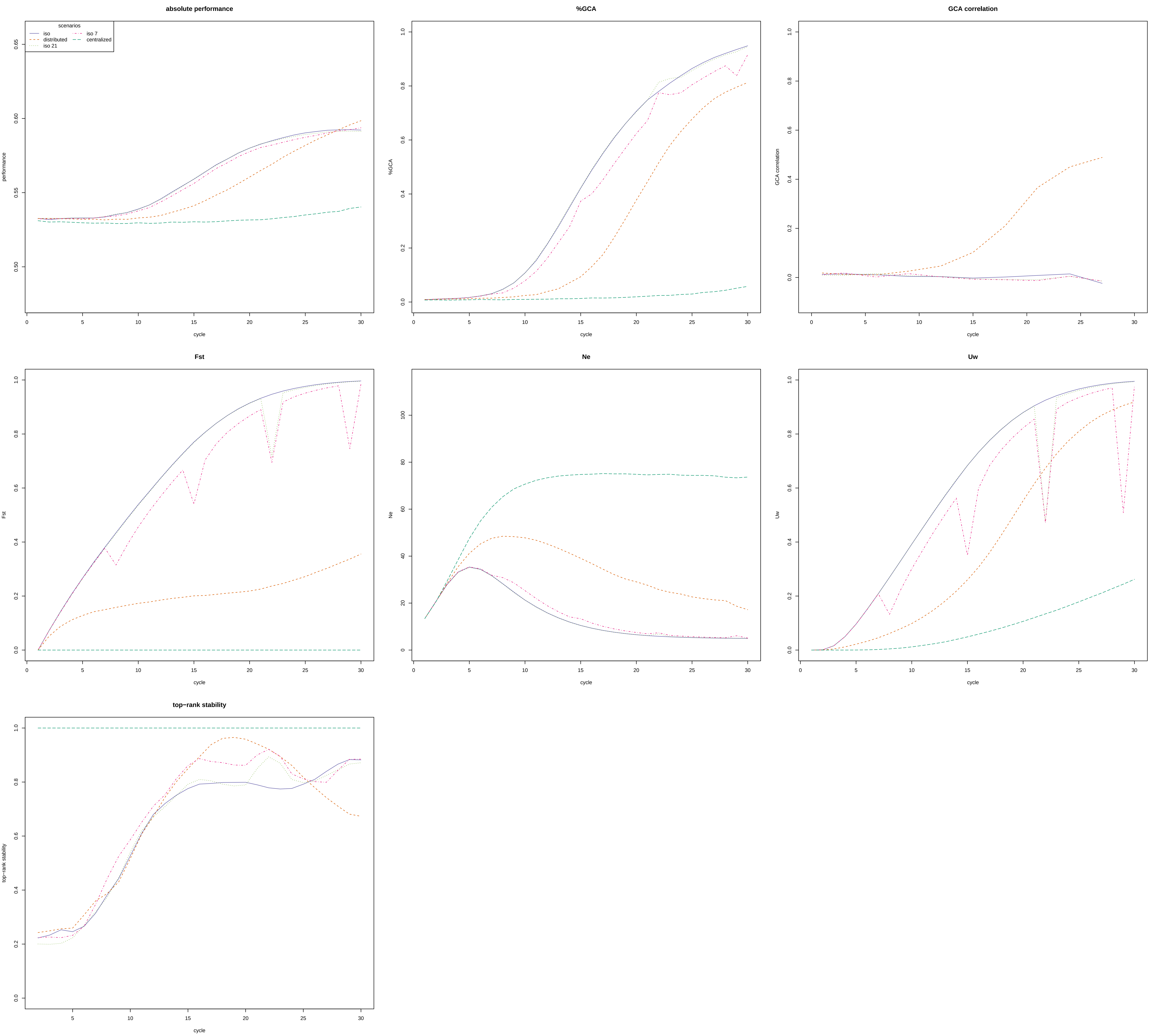
absolute performance
scenarios
iso
distributed
iso 21
iso 7
centralized
performance
cycle
%GCA
GCA correlation
Fst
Ne
Uw
top−rank stability

k = 13

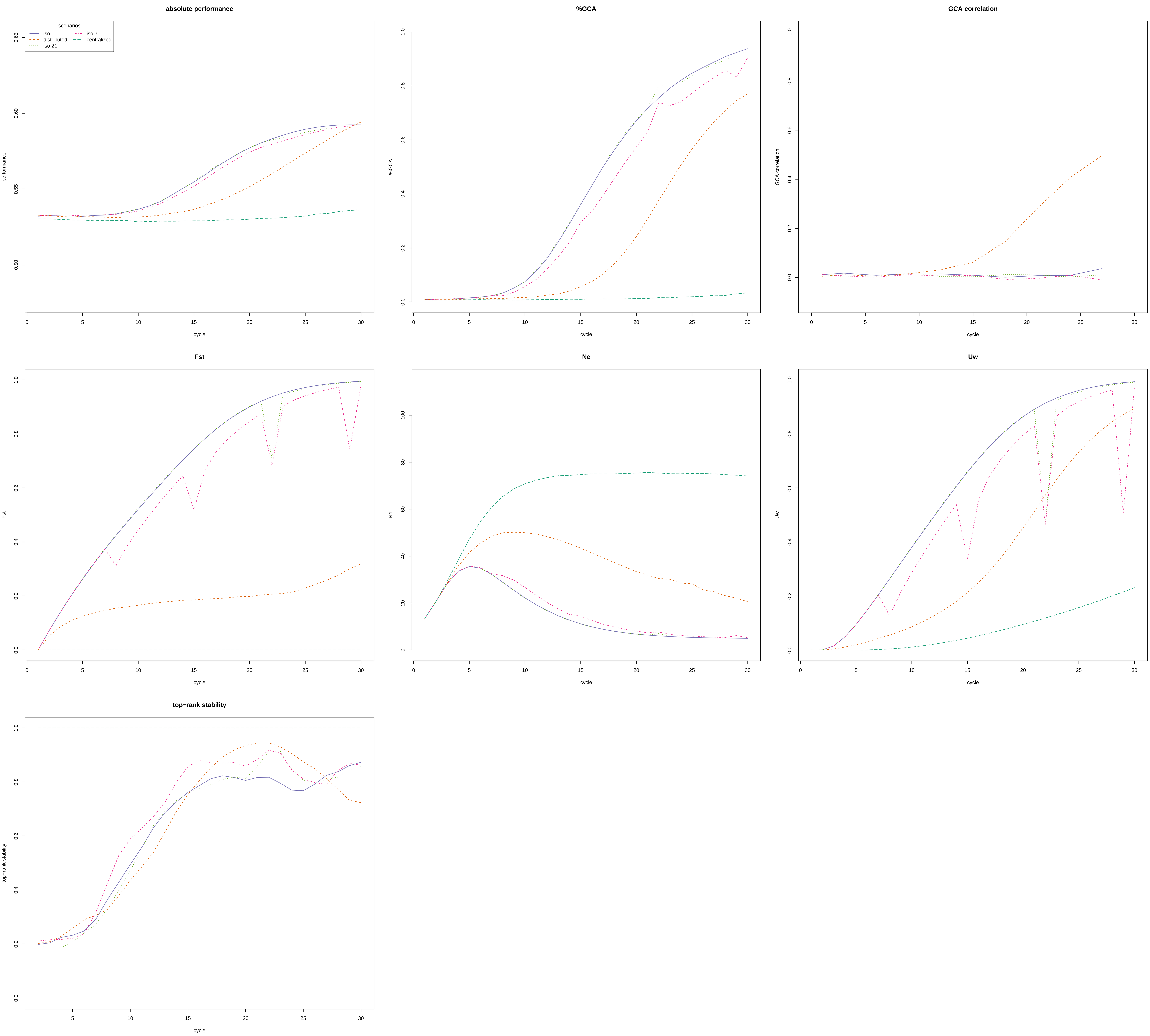

absolute performance
scenarios
iso
distributed
iso 21
iso 7
centralized
performance
cycle
%GCA
GCA correlation
Fst
Ne
Uw
top−rank stability

k = 14

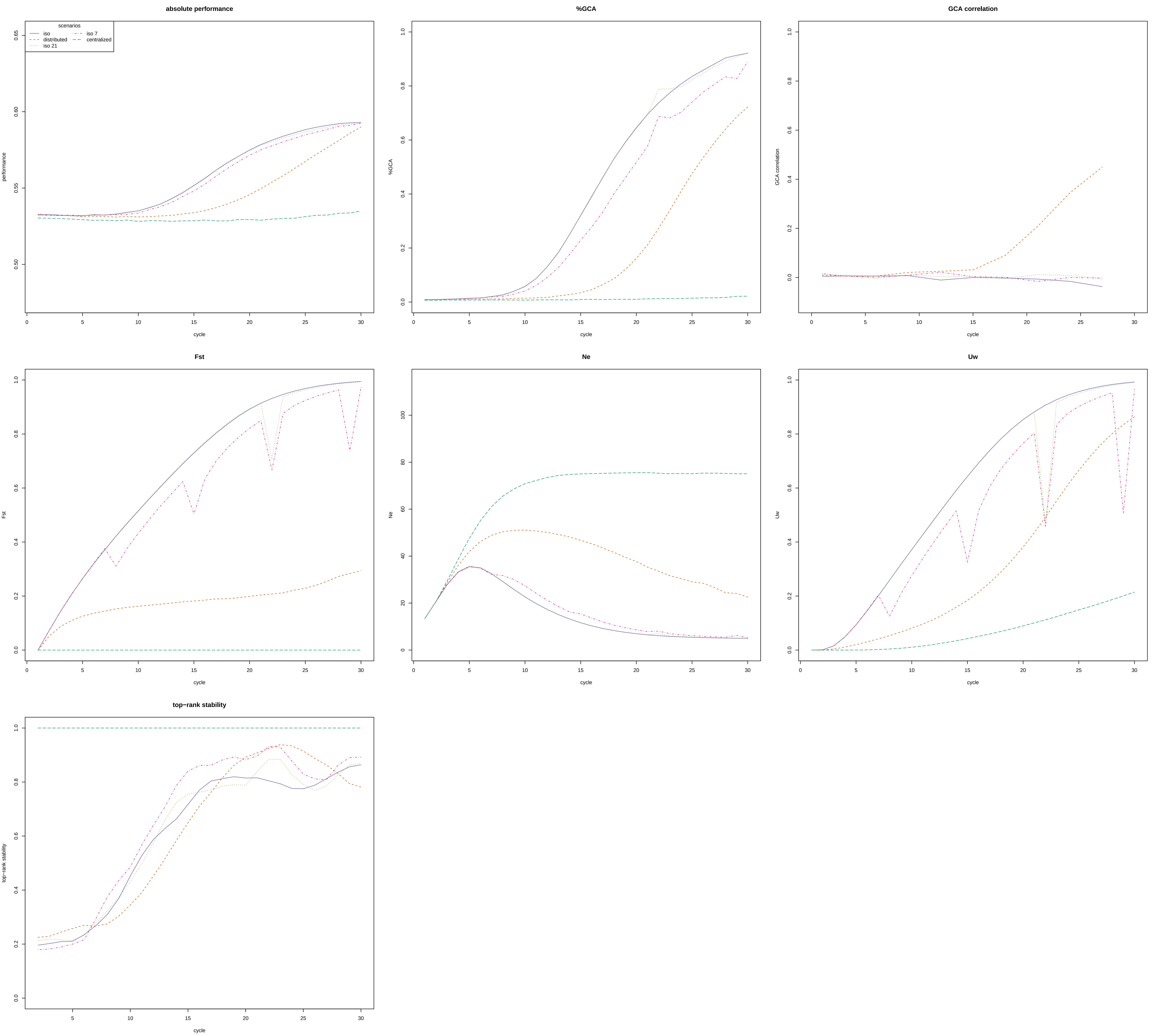
absolute performance
scenarios
iso
distributed
iso 21
iso 7
centralized
performance
cycle
%GCA
GCA correlation
Fst
Ne
Uw
top−rank stability

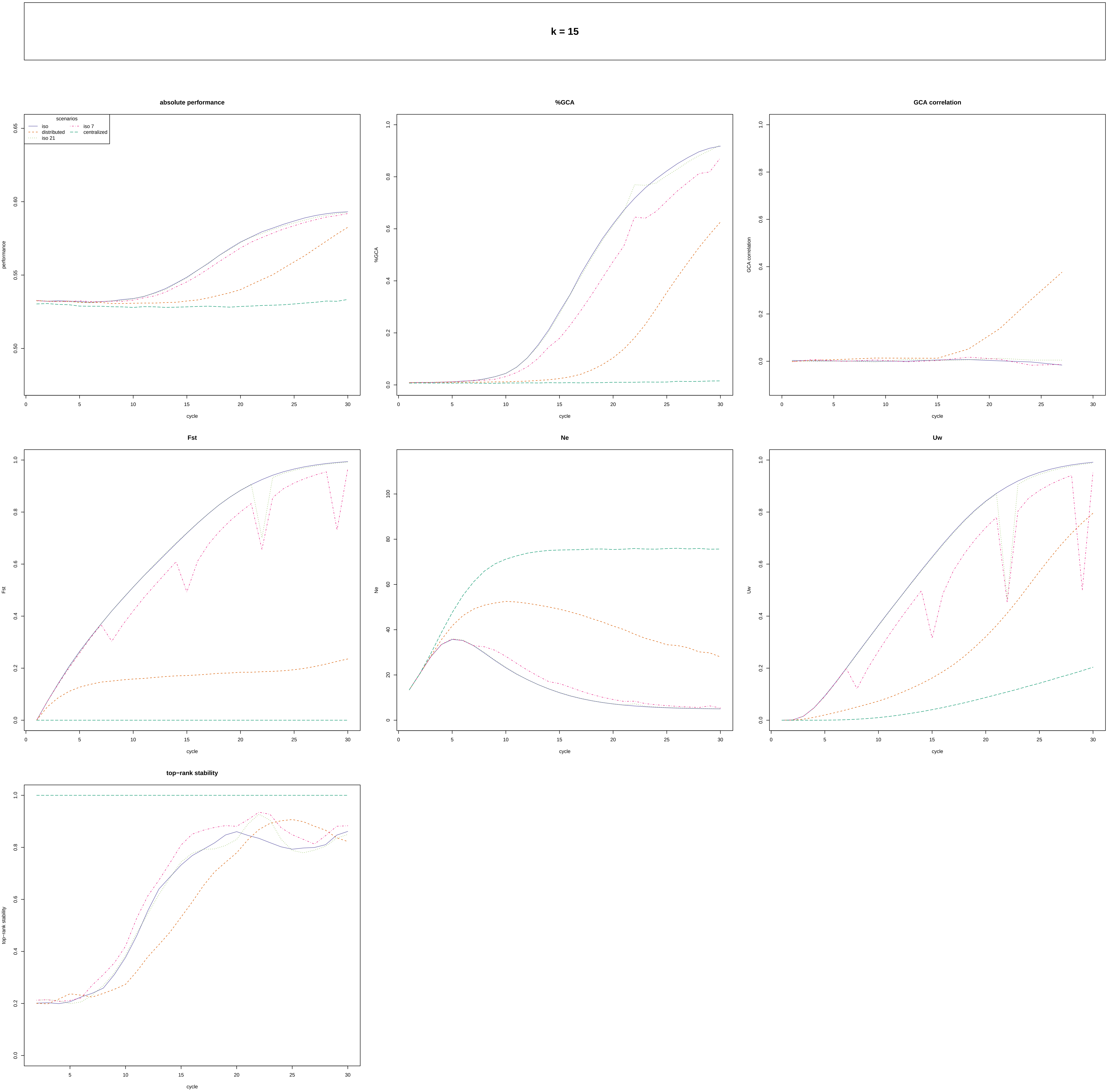
k = 15
absolute performance
scenarios
iso
distributed
iso 21
iso 7
centralized
performance
cycle
%GCA
GCA correlation
Fst
Ne
Uw
top−rank stability
