# Supplementary material for: Back to the future 2: the implications of germplasm structure on the balance between short- and long-term genetic gain in a changing target population of environments
Source: G3 (Bethesda). 2026 Feb 19;16(4):jkag044. doi: 10.1093/g3journal/jkag044 (PMC13042289; doi:10.1093/g3journal/jkag044)
Supplement: jkag044_Supplementary_Data [file jkag044_supplementary_data.zip › File_S2_G3-2025-406286.pdf]

k = 1

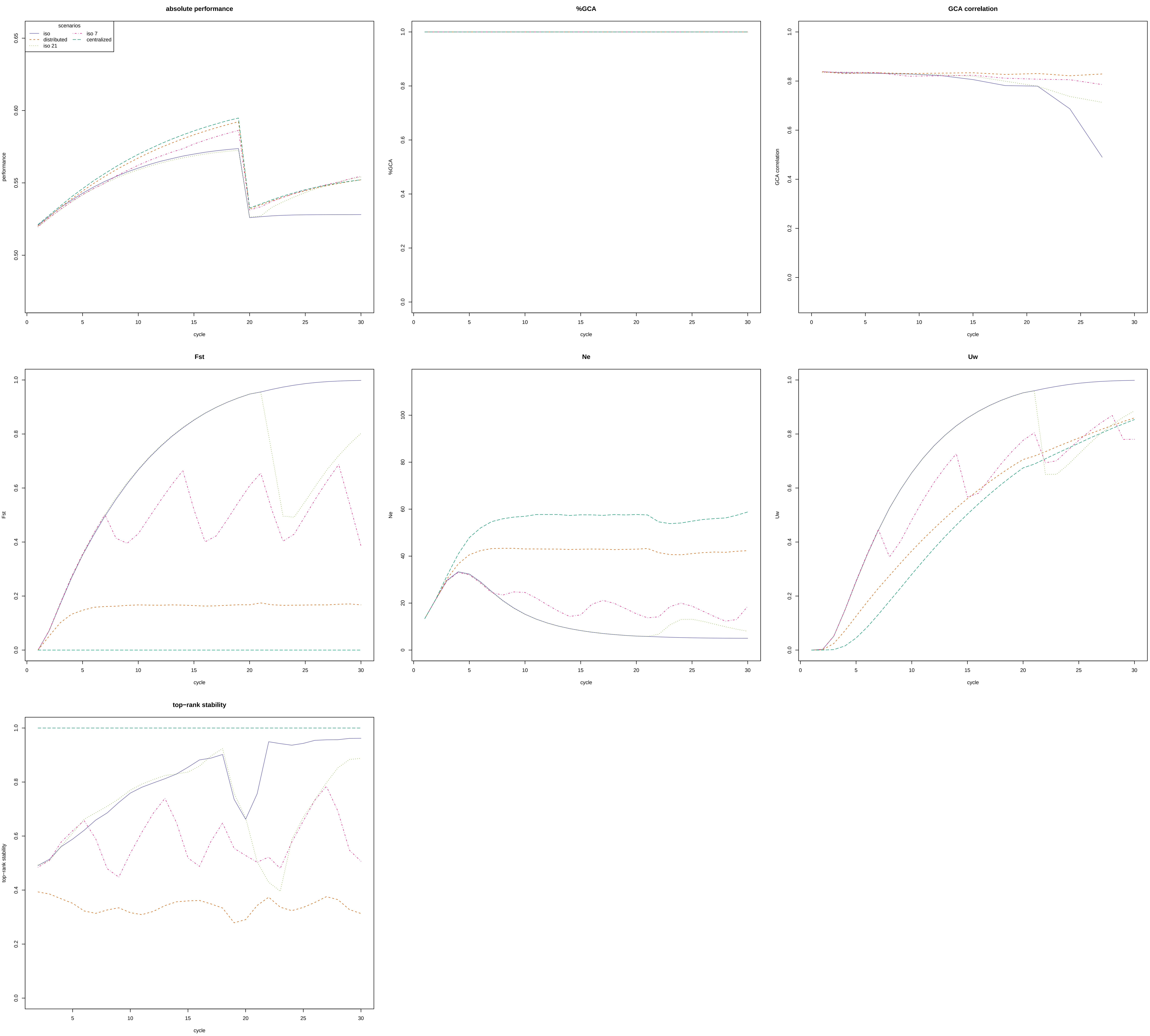
absolute performance
scenarios
iso
distributed
iso 21
iso 7
centralized
performance
cycle
%GCA
GCA correlation
Fst
Ne
Uw
top−rank stability

k = 2

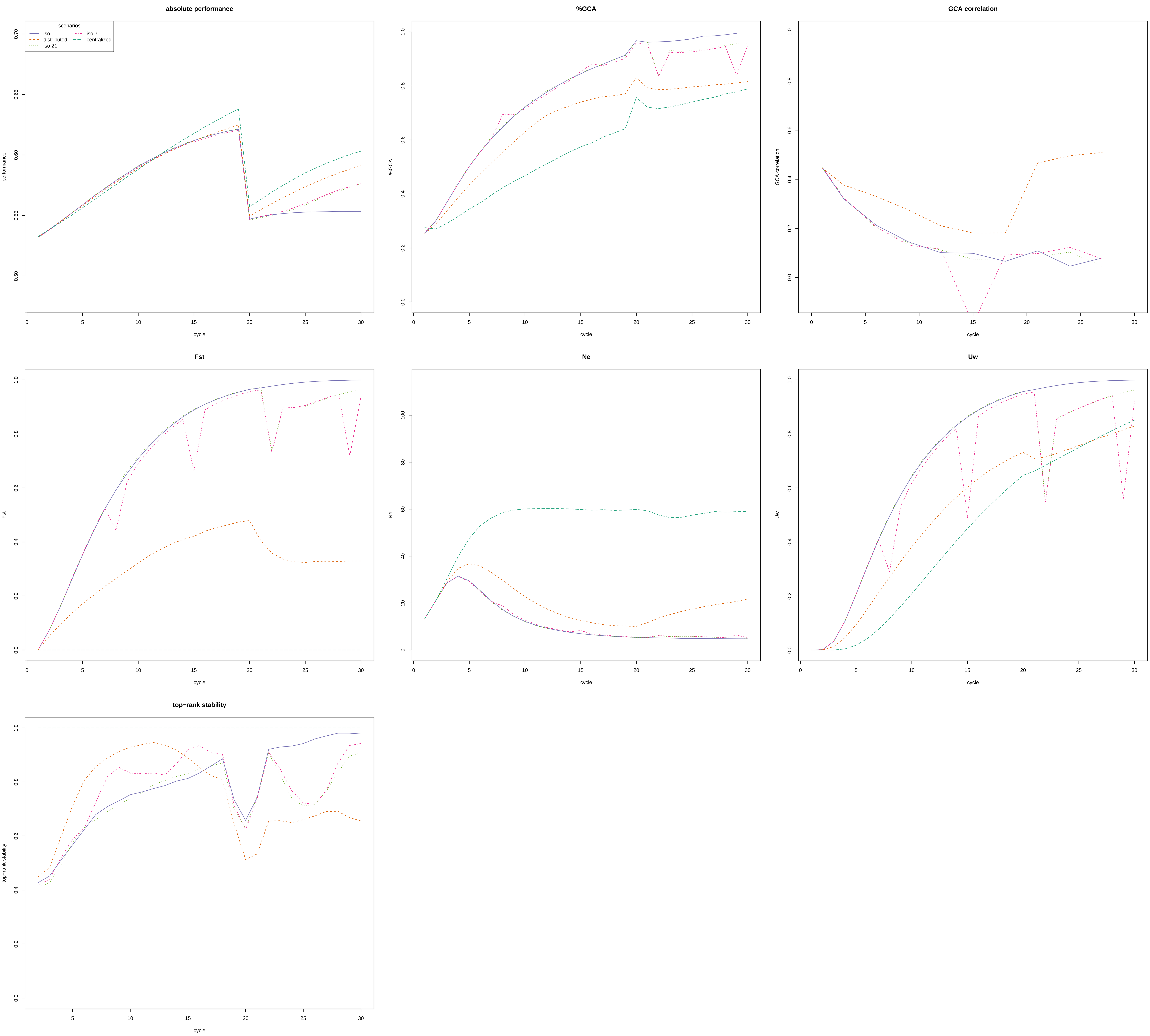
absolute performance
scenarios
iso
distributed
iso 21
iso 7
centralized
performance
cycle
%GCA
GCA correlation
Fst
Ne
Uw
top−rank stability

k = 3

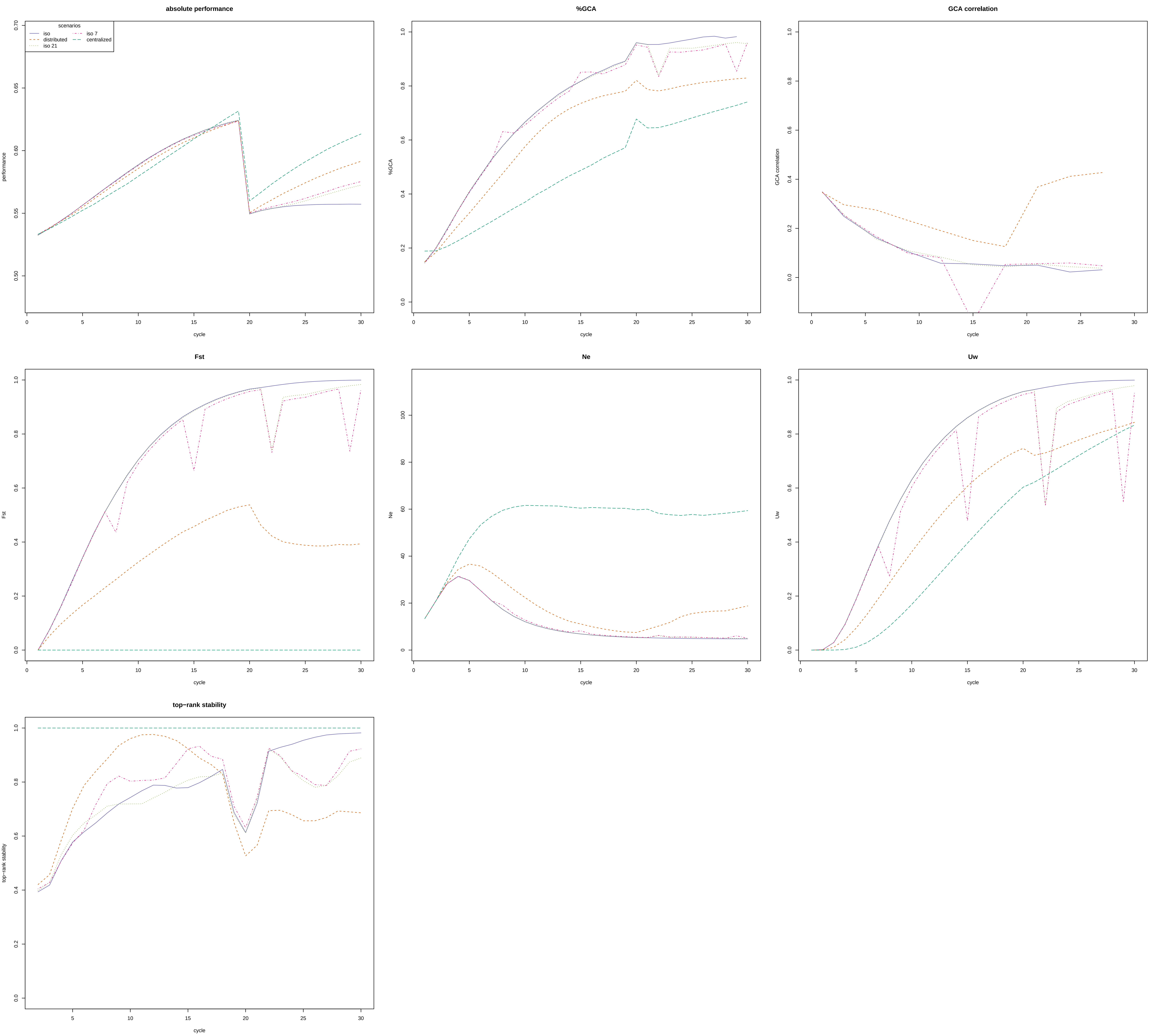
absolute performance
scenarios
iso
distributed
iso 21
iso 7
centralized
performance
cycle
%GCA
%GCA
cycle
GCA correlation
GCA correlation
cycle
Fst
Fst
cycle
Ne
Ne
cycle
Uw
Uw
cycle
top−rank stability
top−rank stability
cycle

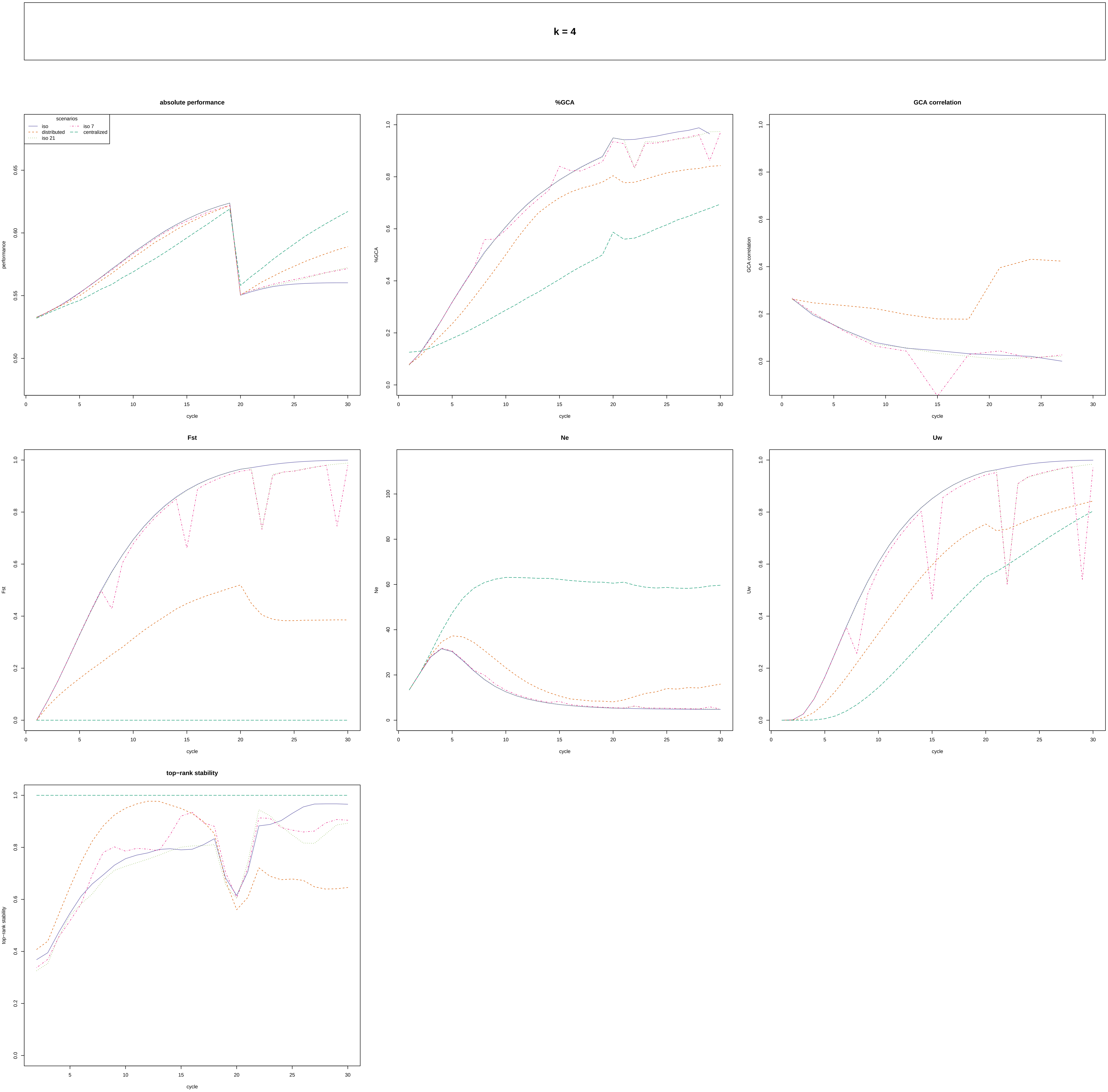
k = 4
absolute performance
scenarios
iso
distributed
iso 21
iso 7
centralized
performance
cycle
%GCA
GCA correlation
Fst
Ne
Uw
top−rank stability

k = 5

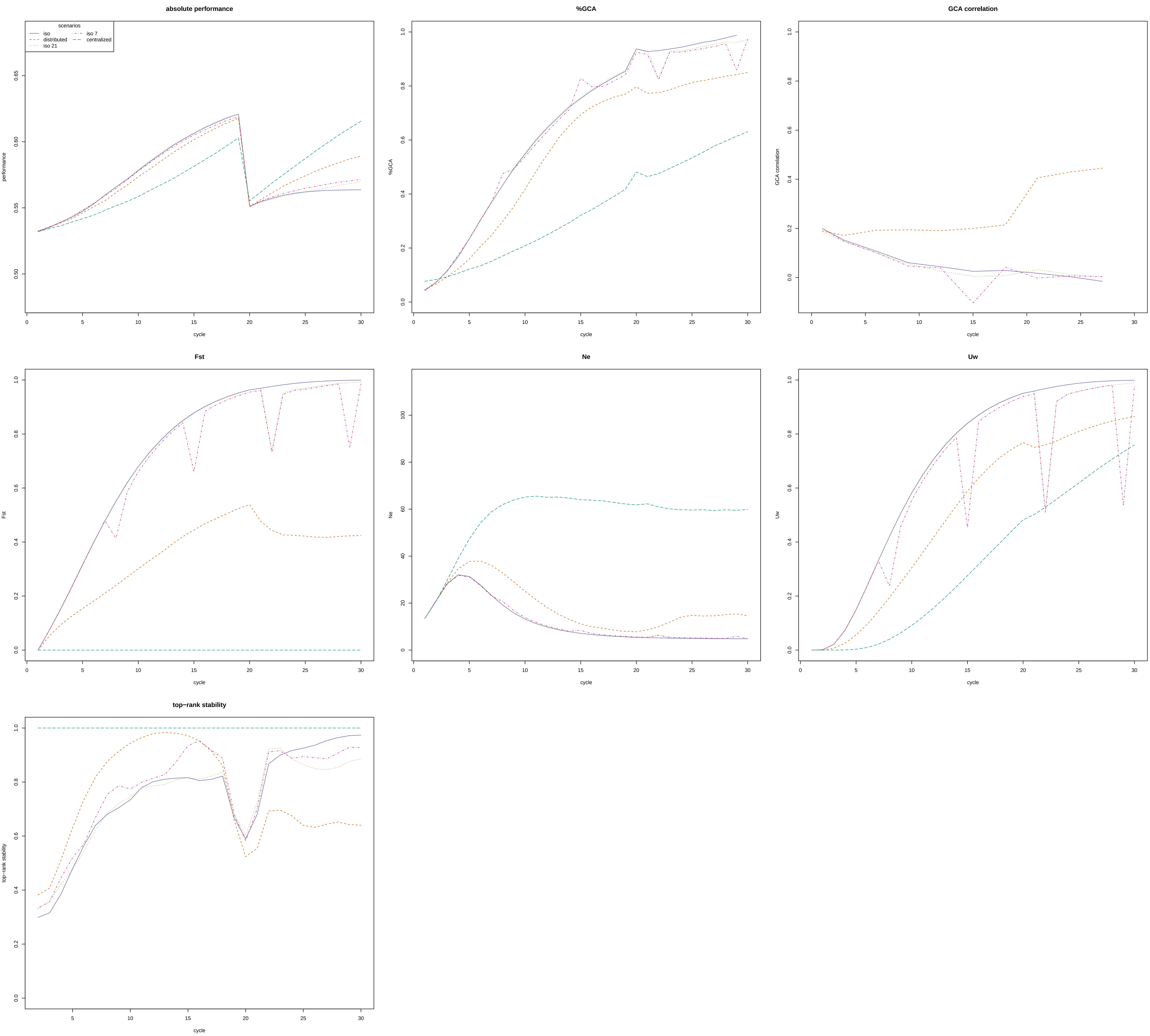
absolute performance
scenarios
iso
distributed
iso 21
iso 7
centralized
performance
cycle
%GCA
%GCA
cycle
GCA correlation
GCA correlation
cycle
Fst
Fst
cycle
Ne
Ne
cycle
Uw
Uw
cycle
top−rank stability
top−rank stability
cycle

k = 6

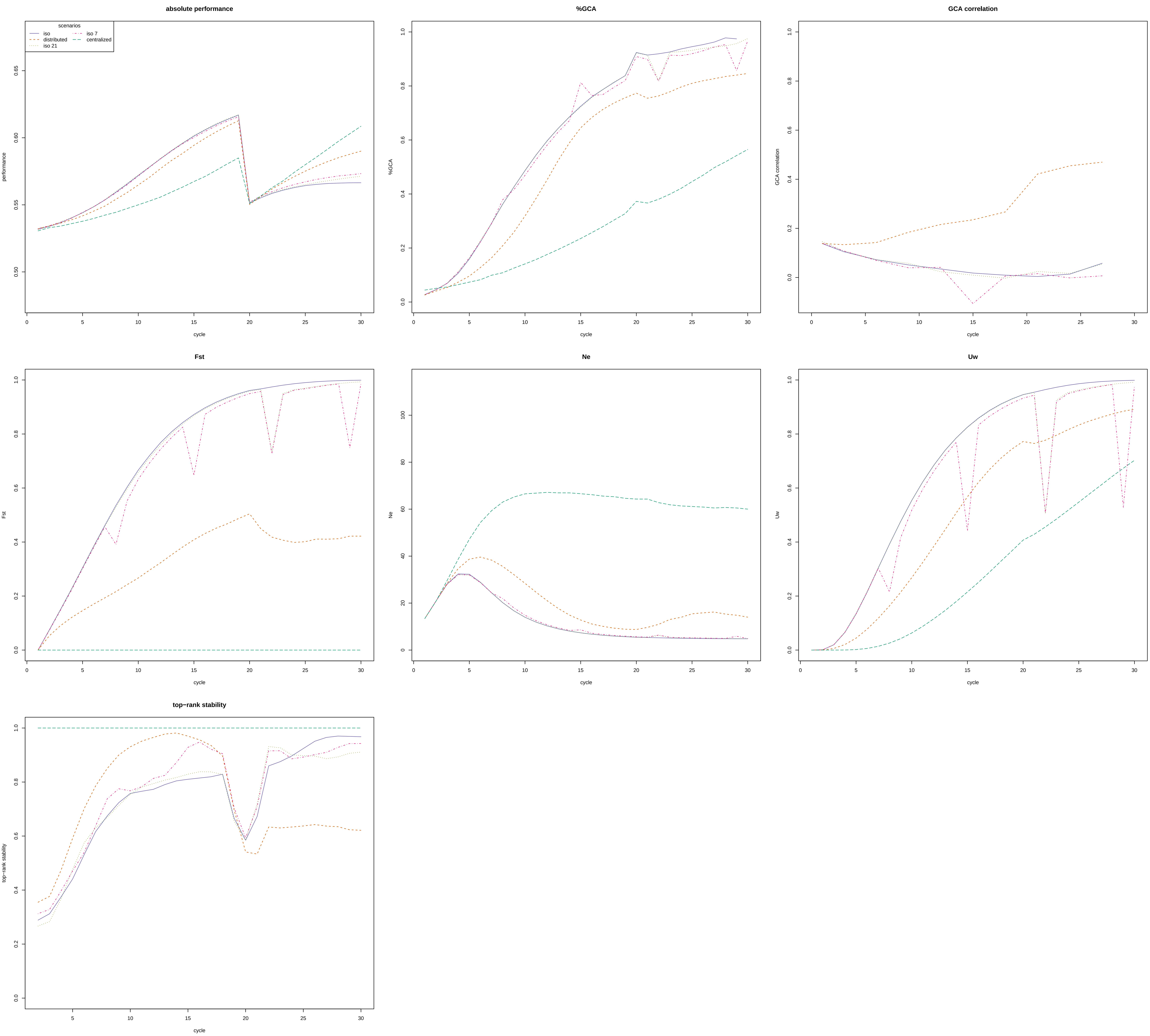

absolute performance
scenarios
iso
distributed
iso 21
iso 7
centralized
performance
cycle
%GCA
GCA correlation
Fst
Ne
Uw
top−rank stability

k = 7

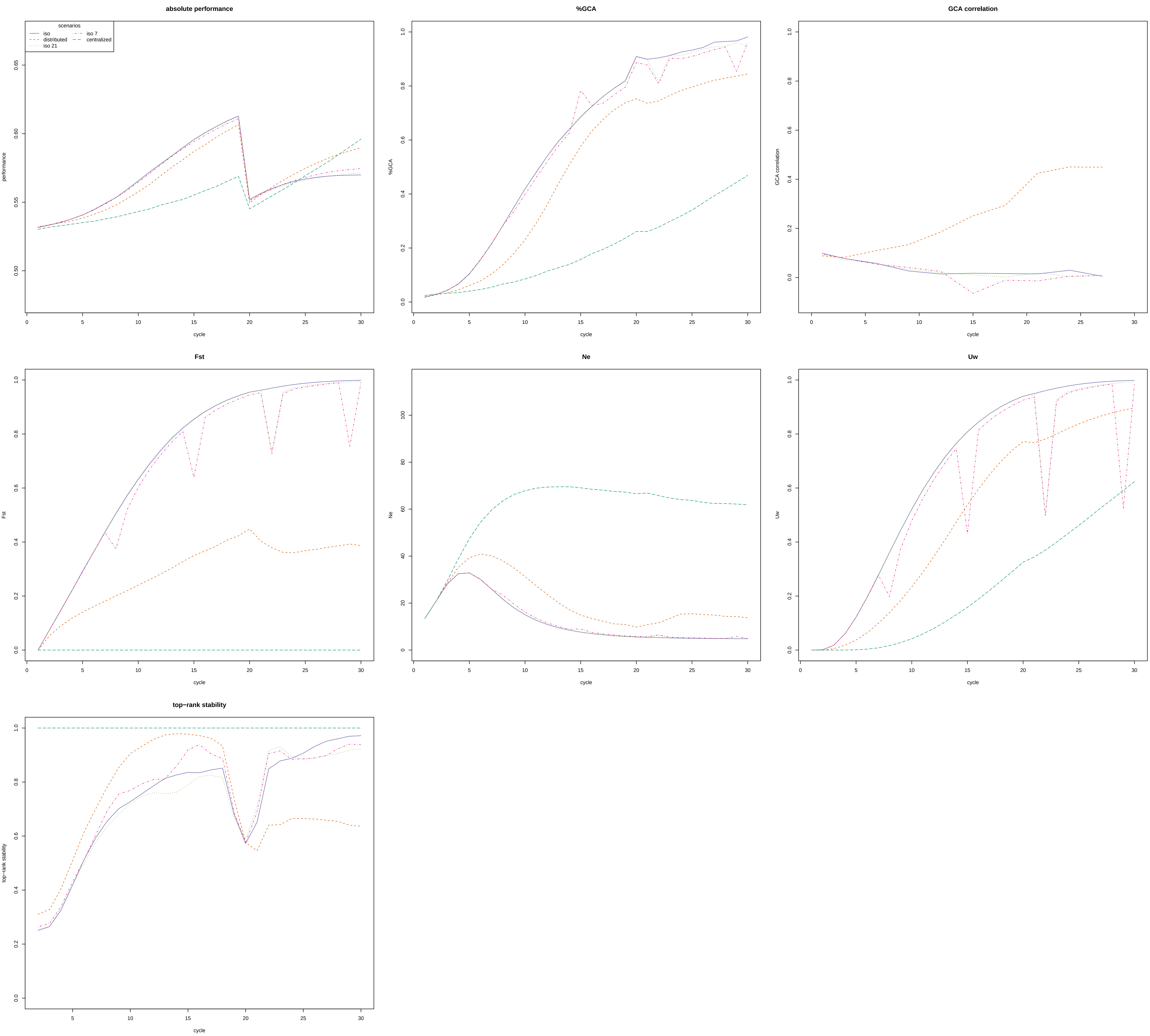
absolute performance
scenarios
iso
distributed
iso 21
iso 7
centralized
performance
cycle
%GCA
%GCA
cycle
GCA correlation
GCA correlation
cycle
Fst
Fst
cycle
Ne
Ne
cycle
Uw
Uw
cycle
top−rank stability
top−rank stability
cycle

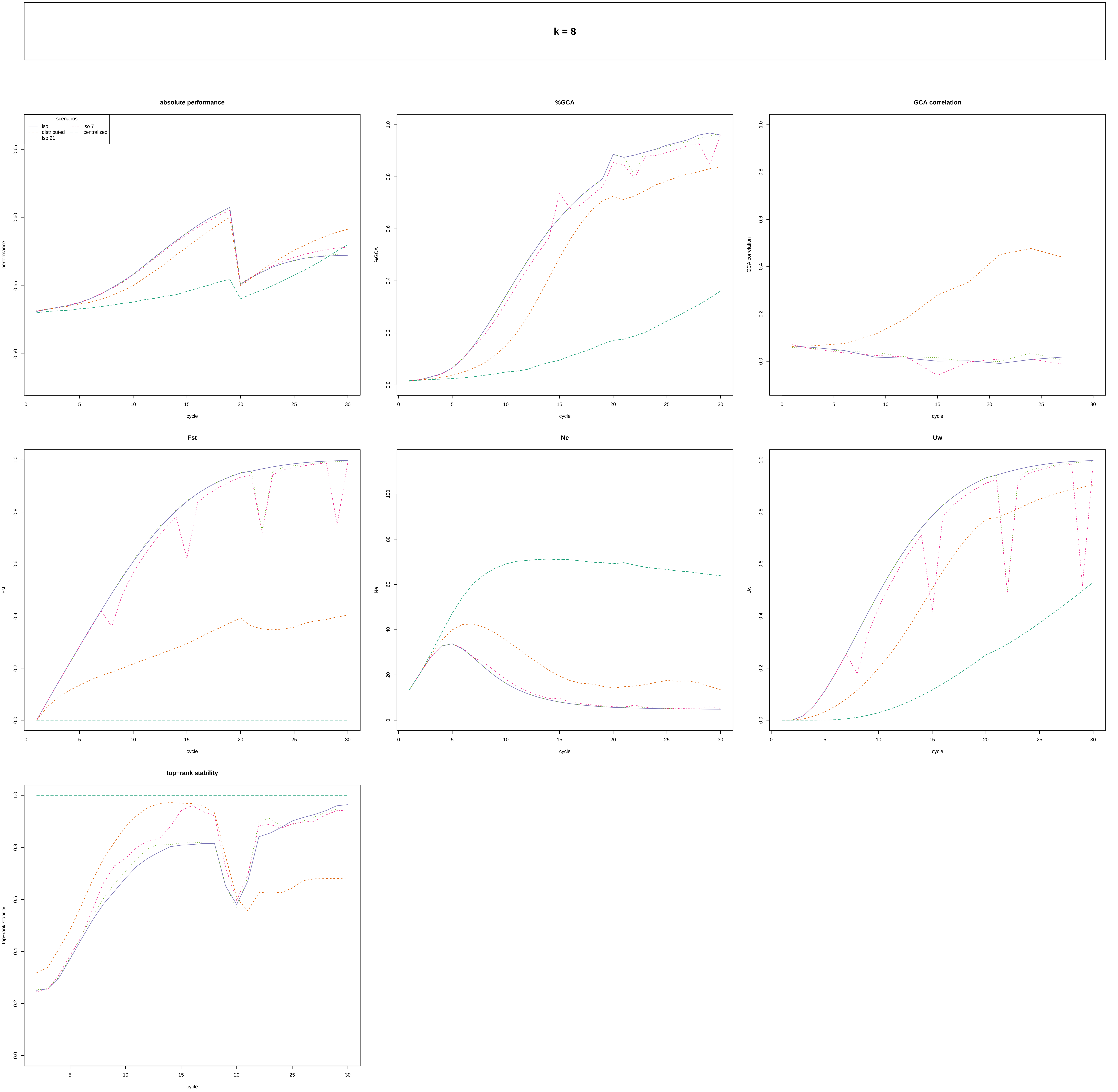
k = 8
absolute performance
performance
cycle
scenarios
iso
distributed
iso 21
iso 7
centralized
%GCA
GCA correlation
Fst
Ne
Uw
top−rank stability

k = 9

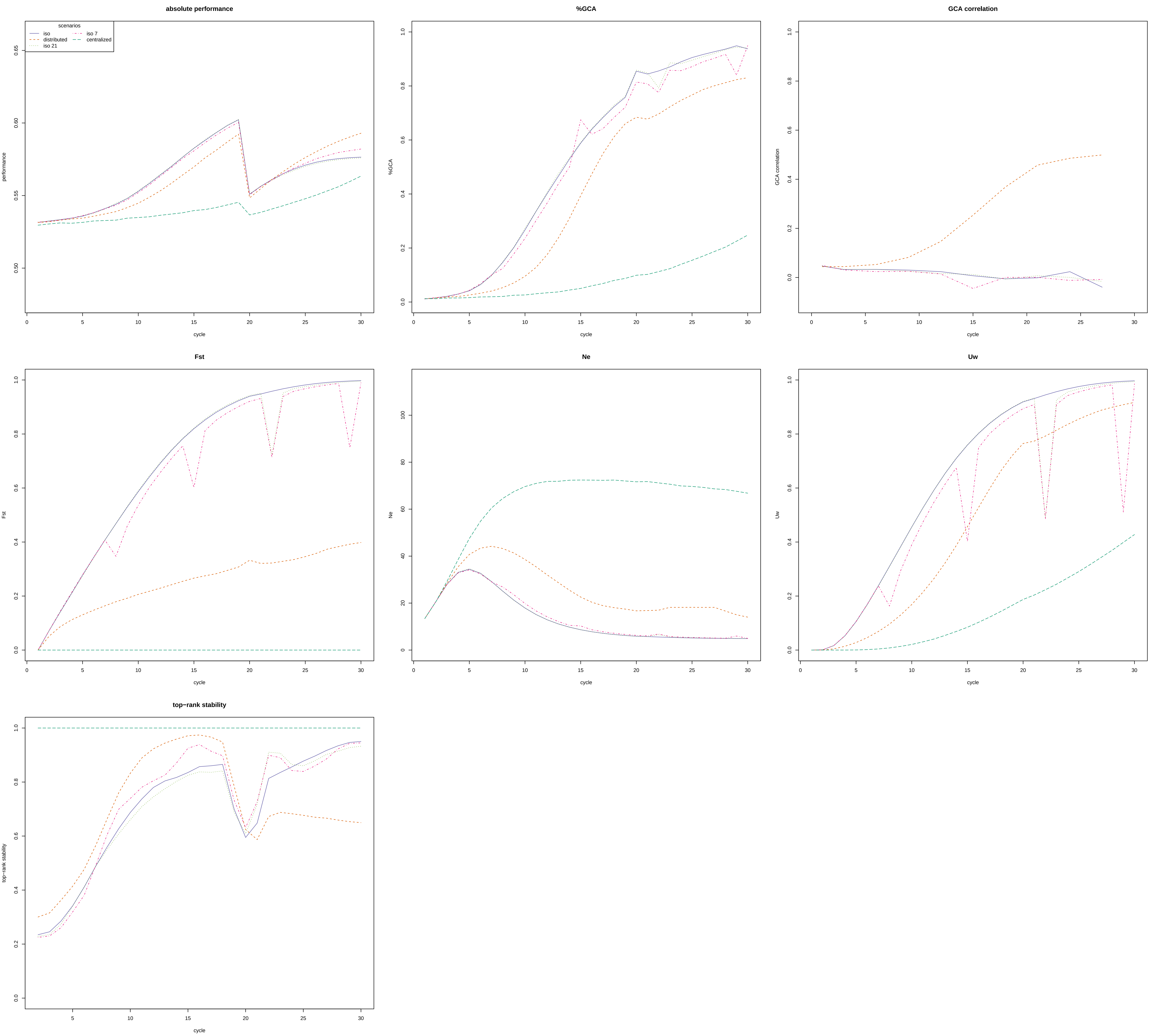

absolute performance
scenarios
iso
distributed
iso 21
iso 7
centralized
performance
cycle
%GCA
GCA correlation
Fst
Ne
Uw
top−rank stability

k = 10

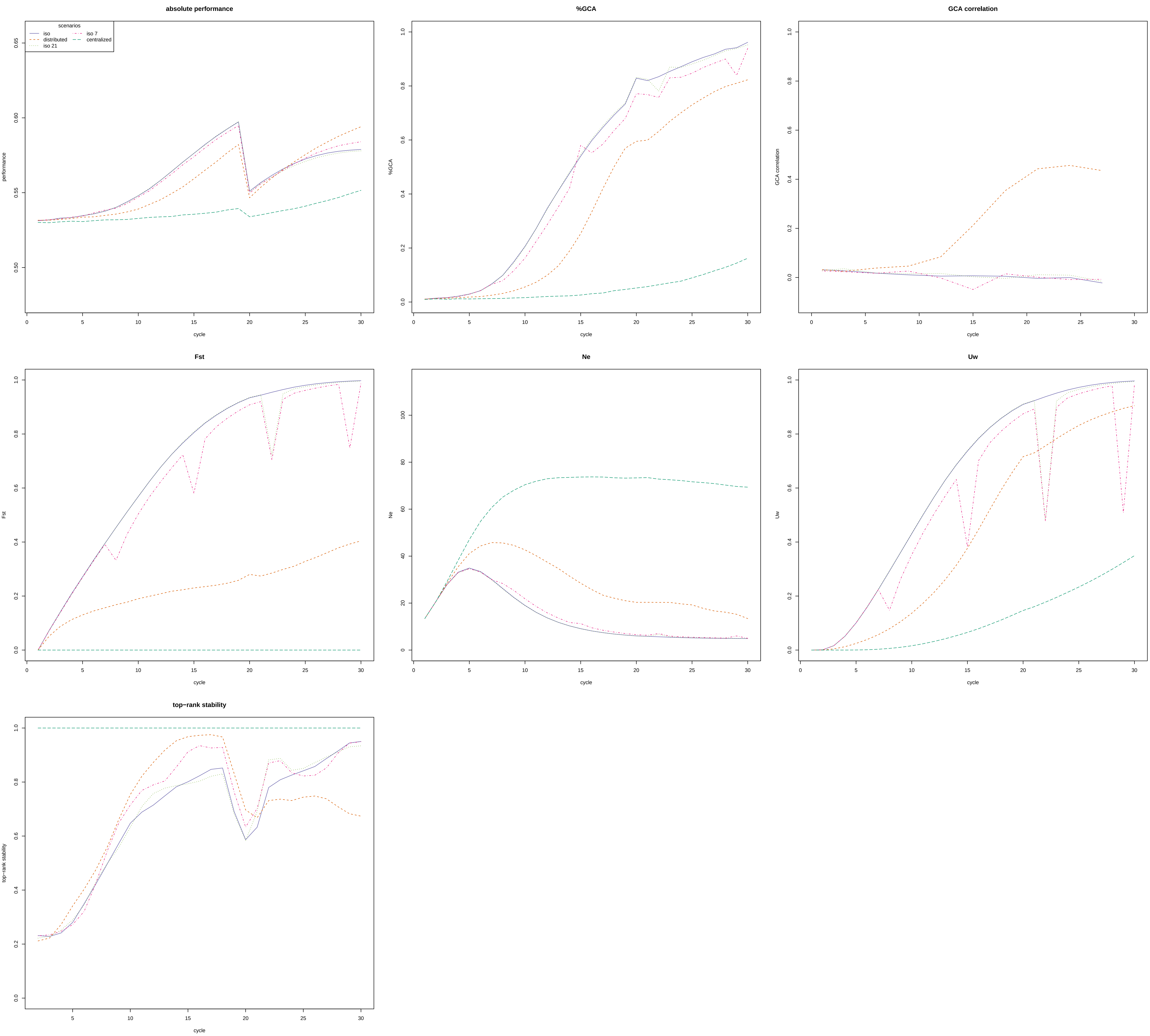
absolute performance
scenarios
iso
distributed
iso 21
iso 7
centralized
performance
cycle
%GCA
GCA correlation
Fst
Ne
Uw
top−rank stability

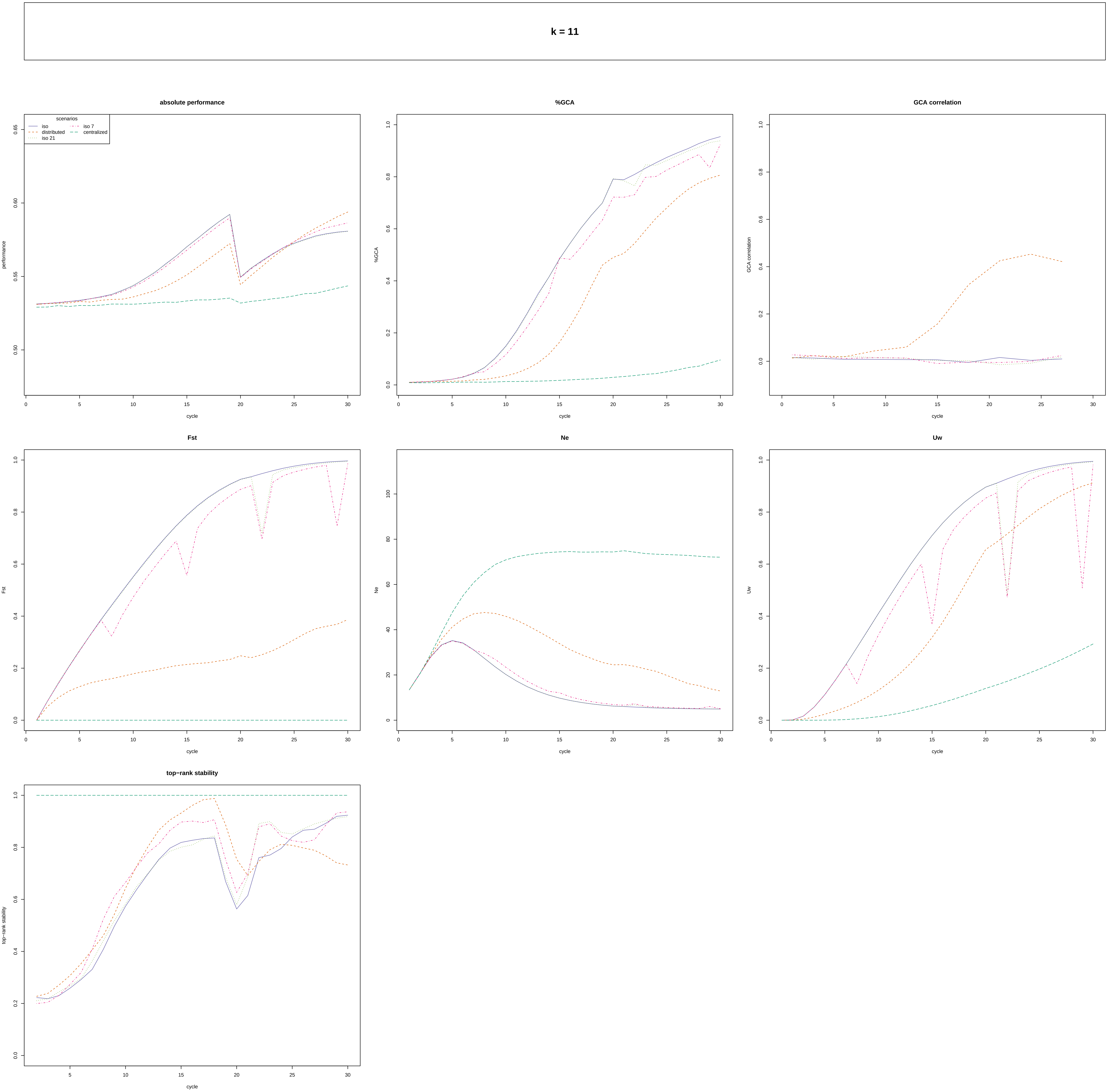

k = 11
absolute performance
scenarios
iso
distributed
iso 21
iso 7
centralized
performance
cycle
%GCA
GCA correlation
Fst
Ne
Uw
top−rank stability

k = 12

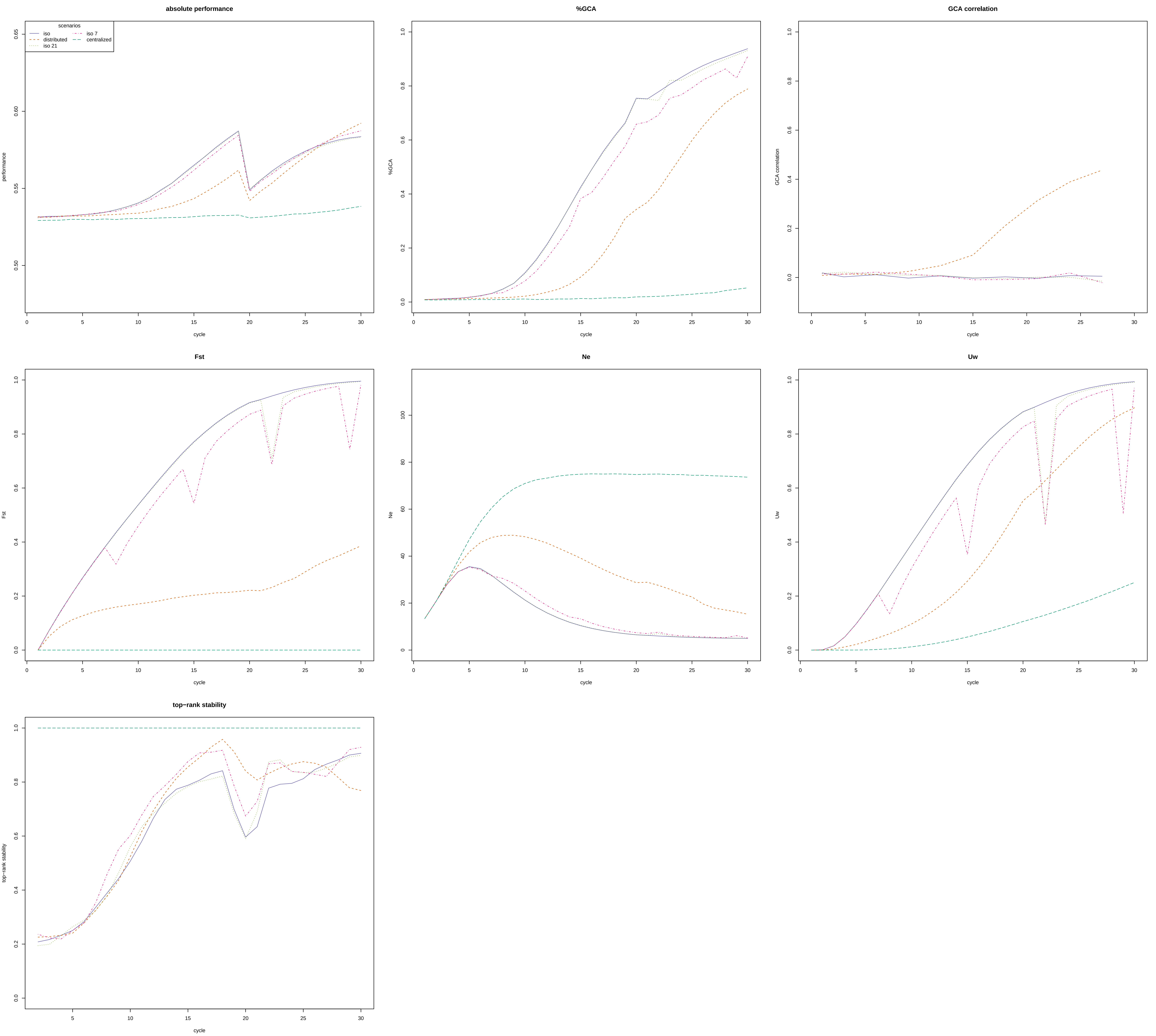
absolute performance
scenarios
iso
distributed
iso 21
iso 7
centralized
performance
cycle
%GCA
%GCA
cycle
GCA correlation
GCA correlation
cycle
Fst
Fst
cycle
Ne
Ne
cycle
Uw
Uw
cycle
top−rank stability
top−rank stability
cycle

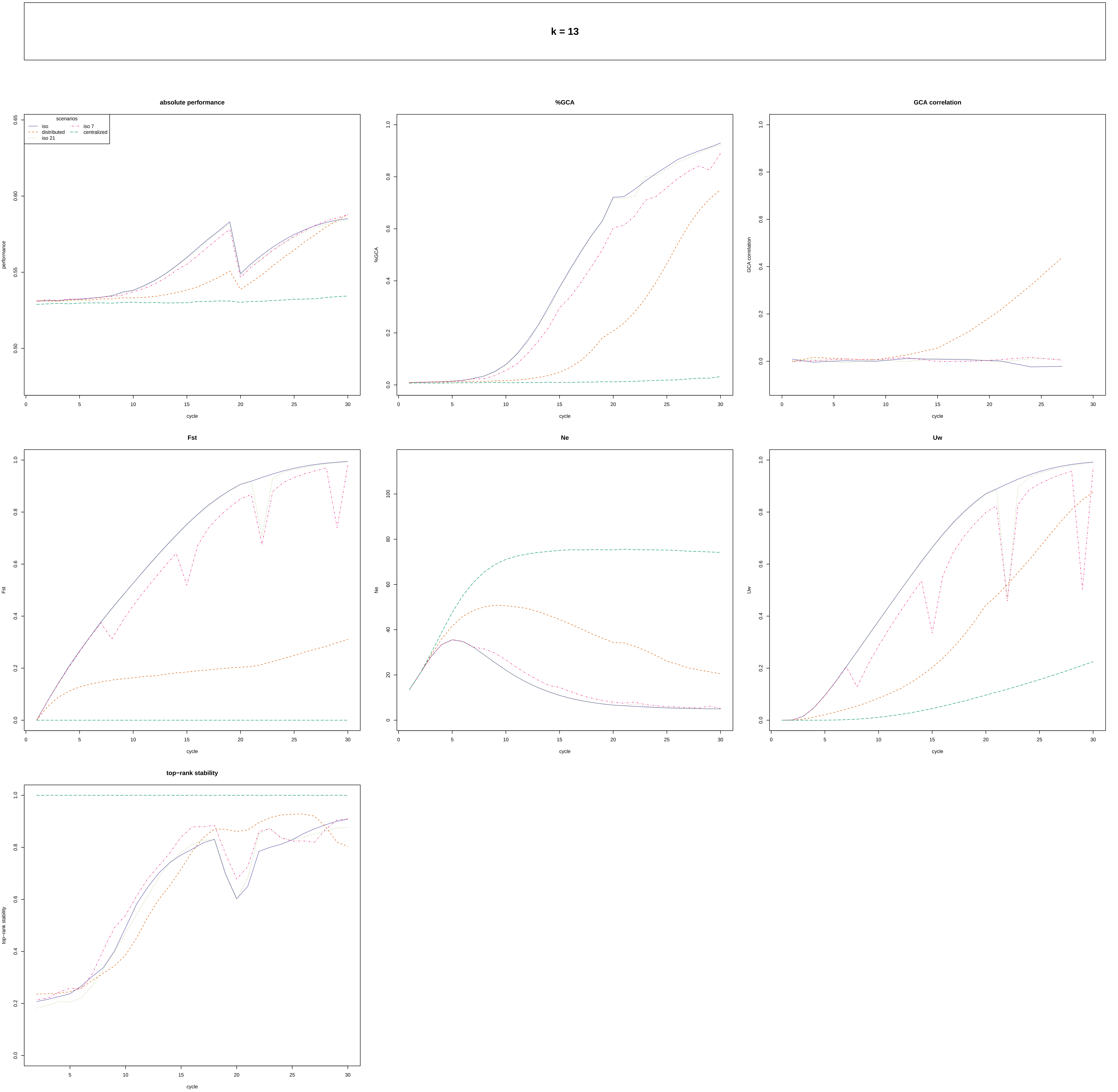
k = 13
absolute performance
scenarios
iso
distributed
iso 21
iso 7
centralized
performance
cycle
%GCA
cycle
GCA correlation
cycle
Fst
cycle
Ne
cycle
Uw
cycle
top−rank stability
cycle

k = 14

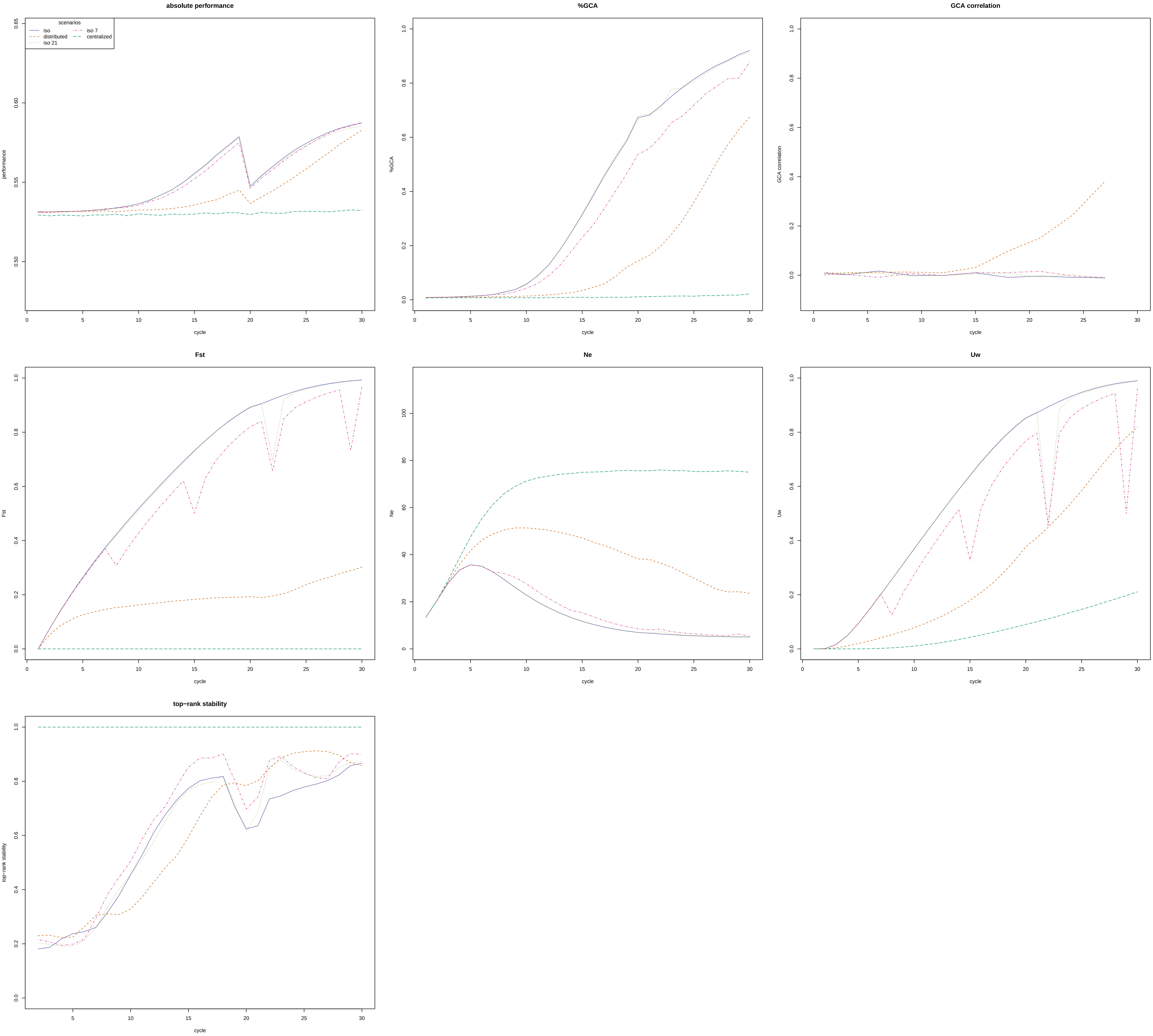
absolute performance
scenarios
iso
distributed
iso 21
iso 7
centralized
performance
cycle
%GCA
cycle
GCA correlation
GCA correlation
cycle
Fst
Fst
cycle
Ne
Ne
cycle
Uw
Uw
cycle
top−rank stability
top−rank stability
cycle

k = 15

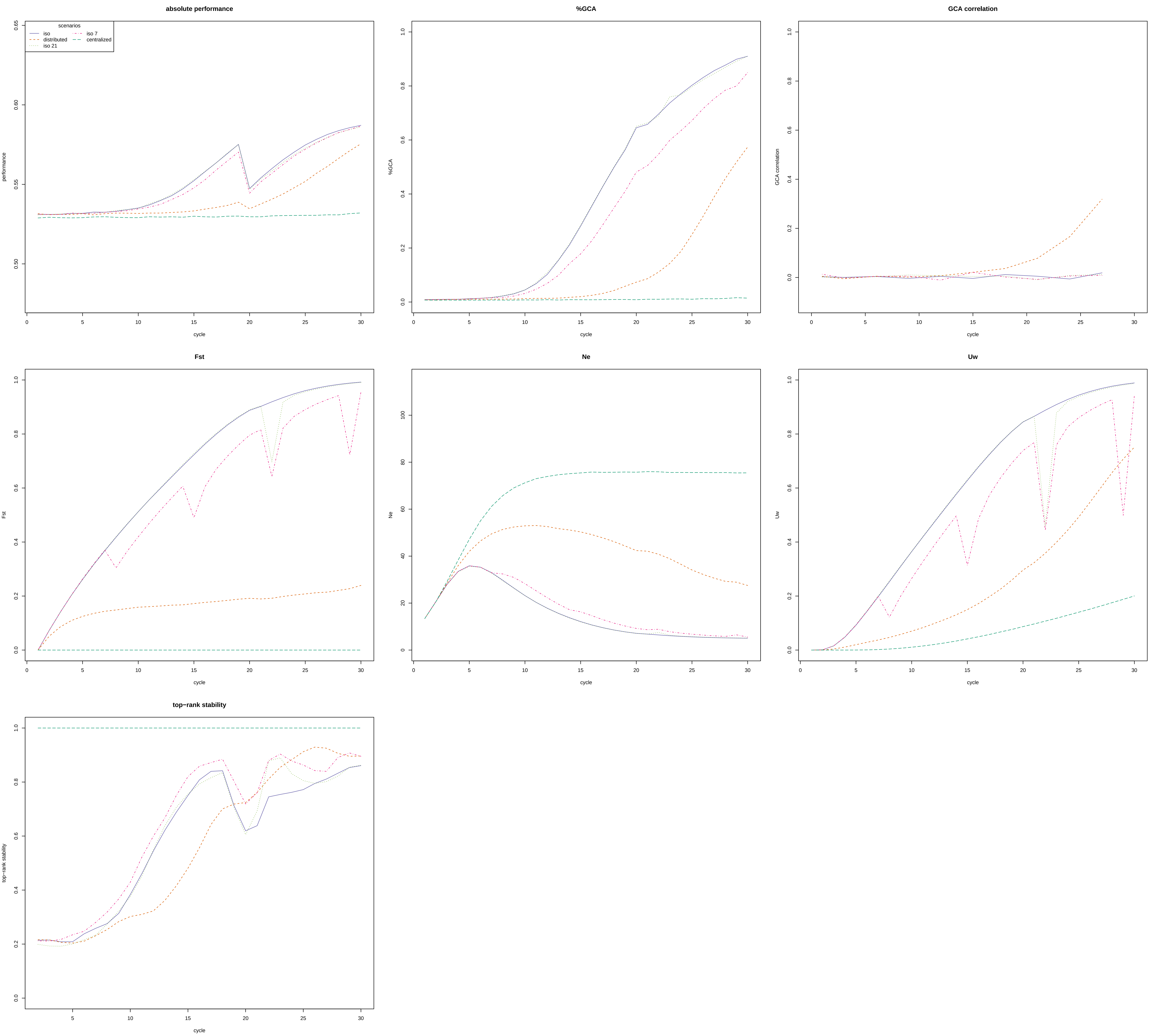
absolute performance
performance
cycle
scenarios
iso
distributed
iso 21
iso 7
centralized
0.50
0.55
0.60
0.65
%GCA
GCA correlation
Fst
Ne
Uw
top−rank stability
0.0
0.2
0.4
0.6
0.8
1.0
0
20
40
60
80
100
0
5
10
15
20
25
30
